# Disease burden attributable to high temperature between 1990 and 2021 in South Asia and Southeast Asia, with projections to 2045

**DOI:** 10.1186/s41182-025-00823-4

**Published:** 2025-10-30

**Authors:** Jingfang Cai, Ahiafor Maxwell, Boda Zhou

**Affiliations:** Beijing Tsinghua Changgung Hospital, School of Clinical Medicine, Tsinghua University, 168 Litang Road, Changping District, Beijing, 102218 China

**Keywords:** High temperature, Global disease burden, South Asia, Southeast Asia, Climate change

## Abstract

**Background:**

Disease burden attributable to extreme high temperature requires more attention amid dramatic climate change, especially in South Asia and Southeast Asia.

**Methods:**

We analyzed comprehensive estimates from the GBD 2021 Study, examining mortality and disability-adjusted life years (DALYs) across 369 diseases and 88 risk factors. This study employed joinpoint regression analysis and Age-Period-Cohort modeling to examine time trends from 1990 to 2021 and projected disease burden up to 2045 by incorporating demographic forecasts and a Bayesian Age-Period-Cohort model.

**Results:**

South Asia and Southeast Asia contributed more than half of the global death number attributed to high temperature. In 2021, South Asia recorded 209,537 deaths and Southeast Asia recorded 32,230 deaths attributed to high temperatures. In South Asia and Southeast Asia, Pakistan bore the highest number and rate of deaths attributed to high temperature. The population above 55 and below 5 years in South Asia and Southeast Asia experienced higher disease burden attributed to high temperature. The leading cause of ASMR attributed to high temperature in South Asia and Southeast Asia was non-communicable diseases. Population growth and aging were the main drivers of ASMR increases in South Asia and Southeast Asia, while epidemiological changes contributed to a reduction in ASMR. Deaths attributed to high temperatures in South and Southeast Asia are projected to rise until 2045, with South Asia exceeding 400,000 and Southeast Asia approaching 100,000 deaths in 2045.

**Conclusions:**

This study highlights the urgent need for region-specific, gender-specific and age-specific interventions to reduce high temperature-related disease burden in South Asia and Southeast Asia.

**Supplementary Information:**

The online version contains supplementary material available at 10.1186/s41182-025-00823-4.

## Introduction

The surge in the impact of climate change on human health has emerged as one of the most pressing challenges of the twenty-first century. Rising global temperatures pose a significant threat to population health, particularly in vulnerable regions. According to reports by the Intergovernmental Panel on Climate Change, global surface temperatures have increased by approximately 1.1 °C above pre-industrial levels [1], setting in motion adverse consequences for human health. Recent evidence suggests that high temperatures are increasingly linked to escalating emergency department visits, hospitalizations, and mortality rates, with significant rises in cardiovascular and respiratory disease mortality [2, 3]. Globally, non-optimal temperatures account for 5.08 million annual deaths, with mortality rates rising 1.5 times faster in tropical regions [4, 5].

South Asia and Southeast Asia, home to over 2 billion people, are at the forefront of a rapidly escalating global health crisis [6], which have emerged as critical focus points in understanding the health impacts of extremely high temperature. The characteristic unprecedented demographic transitions, population aging and rapid urbanization, coupled with limited healthcare infrastructure and rising temperature, have transformed these regions into epicenters of climate-related health impacts. Furthermore, the socioeconomic disparities across countries and communities exacerbate high temperature related health risks, particularly affecting vulnerable populations such as children under 5 and older adults above 55 years [7].

Previous studies have highlighted South Asia as a global hotspot for heat-related mortality [8]. For instance, the lethal heatwaves of 2022 in Pakistan and India surpassed 50 °C, claiming over 1500 lives, serve as a grim prelude to a future, where extreme heat could reshape regional disease burdens [9]. Yet, while global analyses have quantified temperature-related health risks [10], critical gaps persist in understanding how these burdens manifest inequitably across geographies, generations, and genders in South Asia and Southeast Asia.

In this paper, we leveraged the GBD 2021 study framework to examine time trends and demographic patterns of heat-related disease burden in South Asia and Southeast Asia, as well as employed advanced statistical methodologies, such as joinpoint regression and age-period-cohort modeling, to provide insights into the evolving nature of health impacts caused by high temperature. Our investigation spans three decades of data (1990–2021), enabling us to disentangle the contributions of aging populations, period effects, and cohort-specific vulnerabilities to inform policy formulation and effective resource allocation. In addition, we project the future trajectory of disease burden caused by high temperature to 2045, providing critical insights for policymakers and healthcare planners. Beyond statistics, understanding the disease burden attributable to high temperature in South Asia and Southeast Asia is not merely a regional concern but a global imperative. These regions serve as a microcosm of the broader challenges posed by climate change, offering valuable lessons for mitigating its health impacts worldwide [11, 12].

## Methods

### Overview

We utilized comprehensive estimates of mortality, DALYs and risk factor impacts across 369 diseases and injuries, 88 risk factors, 204 countries and territories provided by the GBD 2021 Study [13, 14]. Data collected from GBD 2021 (https://vizhub.healthdata.org/gbd-results/) were stratified by region, gender, country, and risks. This study adhered to the World Health Organization’s (WHO) guidelines for accurate and transparent data on disease prevalence, incidence, mortality rates and DALYs [15].

### Study data

This study extracted estimates and their corresponding 95% Uncertainty Interval (UI) for 1990–2021 mortality and DALYs in South Asia and Southeast Asia attributable to high temperature in all sexes, all ages and age-standardization groups from the GBD 2021 data. High temperature exposure referred to daily exposure to non-optimal temperatures above the theoretical minimum risk exposure level (TMREL) [16]. The 95% uncertainty intervals (UIs) for all estimates derived from the 25th and 975th order statistics (or equivalently, the 2.5th and 97.5th percentiles) from 1000 draws of the posterior distribution at each step of the burden estimation process [17]. Data were further stratified into twenty 5-year groups with the age of 5 as the boundary aggregated across all the countries in South Asia and Southeast Asia. Data also include the causes of disease burden, among which the level 1 causes of disease are communicable, maternal, neonatal, and nutritional diseases, non-communicable diseases, and injuries, while the level 2 causes consist of more specific categories under these broad groupings, such as cardiovascular diseases and chronic respiratory diseases under non-communicable diseases. The countries in Southeast Asia region includes Cambodia, Indonesia, Lao People’s Democratic Republic, Malaysia, Maldives, Mauritius, Myanmar, Philippines, Seychelles, Sri Lanka, Thailand, Timor-Leste, Viet Nam. The countries in South Asia region includes Bangladesh, Bhutan, India, Nepal, Pakistan [18]. We refer to previous research methods [16, 19] to estimate the disease burden attributable to high temperature in South Asia and Southeast Asia. More detailed methods are described in Appendix 1 (Supplementary methods).

### Joinpoint regression analysis

Joinpoint regression analysis was used to quantify trends in disease incidence or mortality over time [20]. This model employs segmented regression on a log-linear regression model to identify inflection points in the trend. This segmented log-linear model identifies inflection points, where trends in ASR significantly change. Using a grid search algorithm, the optimal number of joinpoints (0–5) was determined by minimizing the mean squared error (MSE) [21]. A Monte Carlo permutation test (9,999 iterations) validated the final model, which computed APC and AAPC with 95% CI [22]. Trends were classified as increasing (APC/AAPC 95% CI > 0), decreasing (95% CI < 0), or stable (95% CI inclusive of 0). More details are described in Appendix 1 (Supplementary methods).

### Age-period-cohort model

The Age-Period-Cohort model(APCM) is an advanced analytical tool that enables the estimation of net drift (overall time trends) and local drift (specific time trends) while disentangling the effects of age, period, and birth cohort-three fundamental dimensions influencing disease patterns [23]. In this study, the APCM used 5-year age groups and 5-year periods. Statistical significance was assessed using the Wald *χ*^*2*^ test. The age effect was represented by age-specific rates, while period and cohort effects were expressed as relative risks compared to a reference period or cohort [24].

### Decomposition analysis

A demographic decomposition analysis partitioned changes in mortality and DALYs caused by high temperature into contributions from population aging, population growth, and epidemiological change, among which epidemiological changes are related to change of age-specific and population-standardized mortality rates, independent of demographic structure [25]. We used Das Gupta’s decomposition method [26], and the detailed calculation process is shown in Appendix 1 (Supplementary methods).

### Statistical analysis and projections

The mortality and DALYs attributed to high temperature were summarized as counts and ASRs (per 100,000 population) with 95% UI. The ASRs per 100,000 people were calculated according to the following formula [27]:

$$\frac{{\sum\nolimits_{i = 1}^{N} {\alpha_{i} W_{i} } }}{{\sum\nolimits_{i = 1}^{N} {W_{i} } }}$$

where *α*_*i*_ is the rate for the age group *i*, and ${\text{W}}_{\text{i}}$ corresponds to the number of individuals in the same age group based on the GBD 2021 standard population. *N* refers to the total number of age groups, [27].

We used a Bayesian Age-Period-Cohort Model (BAPCM) model to project age-standardized mortality rate (ASMR) up to 2045, so as to predict the death burden related to high temperature in South Asia and Southeast Asia in the future. This Bayesian extension of generalized linear models accounts for age, period and cohort dynamics, validated extensively in epidemiological forecasting [28]. All data processing, analysis and visualizations were performed using R 4.3.1 (the R Foundation for Statistical Computing, Vienna, Austria).

## Results

### Regional and global differences

**Fig. 1:**
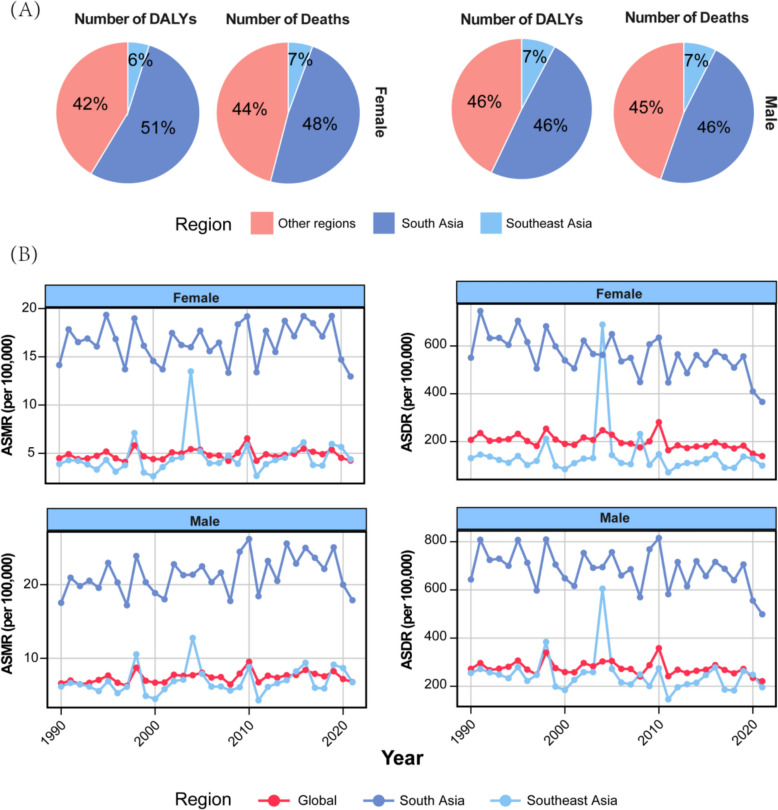
Differences in the **A** number of deaths and DALYs in 2021 and **B** temporal trends in age-standardized mortality rate (ASMR) and age-standardized DALYs rate (ASDR) attributed to high temperature between South Asia, Southeast Asia, and globally

Notably, regardless of gender, the combined death and DALYs in South Asia and Southeast Asia accounted for more than 50% of the global total. Specifically, South Asia alone contributed 46% (male) and 42% (female) of global death number and 46% (male) and 51% (female) of global DALY number, while Southeast Asia added another 7% (similar for male and female) of global death number and 7% (male) and 6% (female) of global DALY number (Fig. 1A). These findings indicate that these two regions together bear a substantial portion of the global disease burden caused by high temperature.

Furthermore, the trends in ASMR and age-standardized disability rate (ASDR) from 1990 to 2021 further highlight regional differences (Fig. 1B). South Asia consistently exhibited ASMRs and ASDRs that were higher than the global averages for both males and females from 1990 to 2021, underscoring the region’s disproportionately high burden of death and disability caused by high temperature. In contrast, Southeast Asia’s ASMRs and ASDRs remained closer to the global averages over the same period, reflecting a moderate yet notable contribution to the global disease burden (Fig. 1B).

### Regional disease burden attributed to high temperature in 2021

**Fig. 2:**
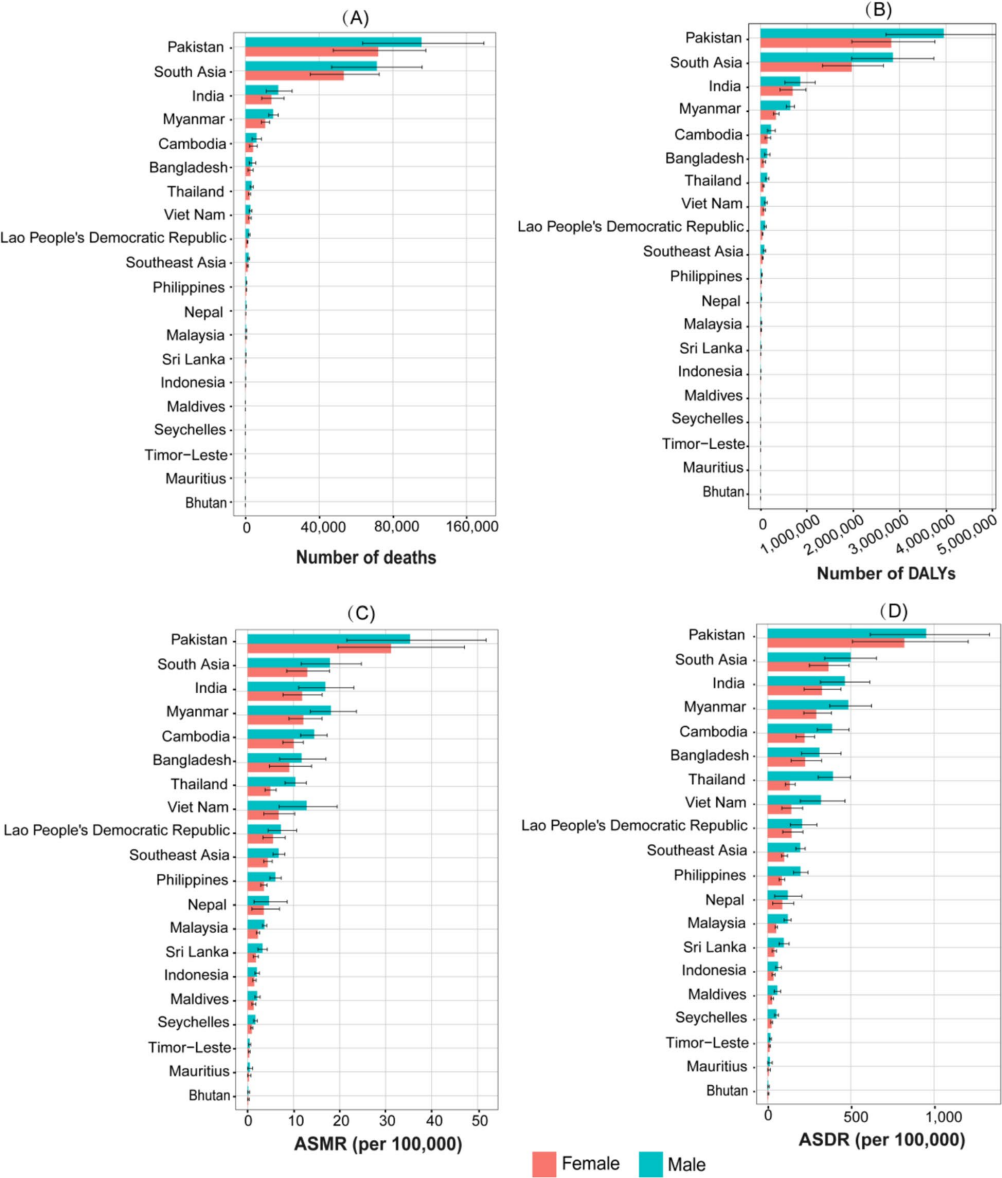
Number of deaths and DALYs (**A**, **B**) and age-standardized mortality rate (ASMR) and age-standardized DALYs rate (ASDR) (**C**, **D**) attributed to high temperature in South Asia and Southeast Asia in 2021

**Fig. 3:**
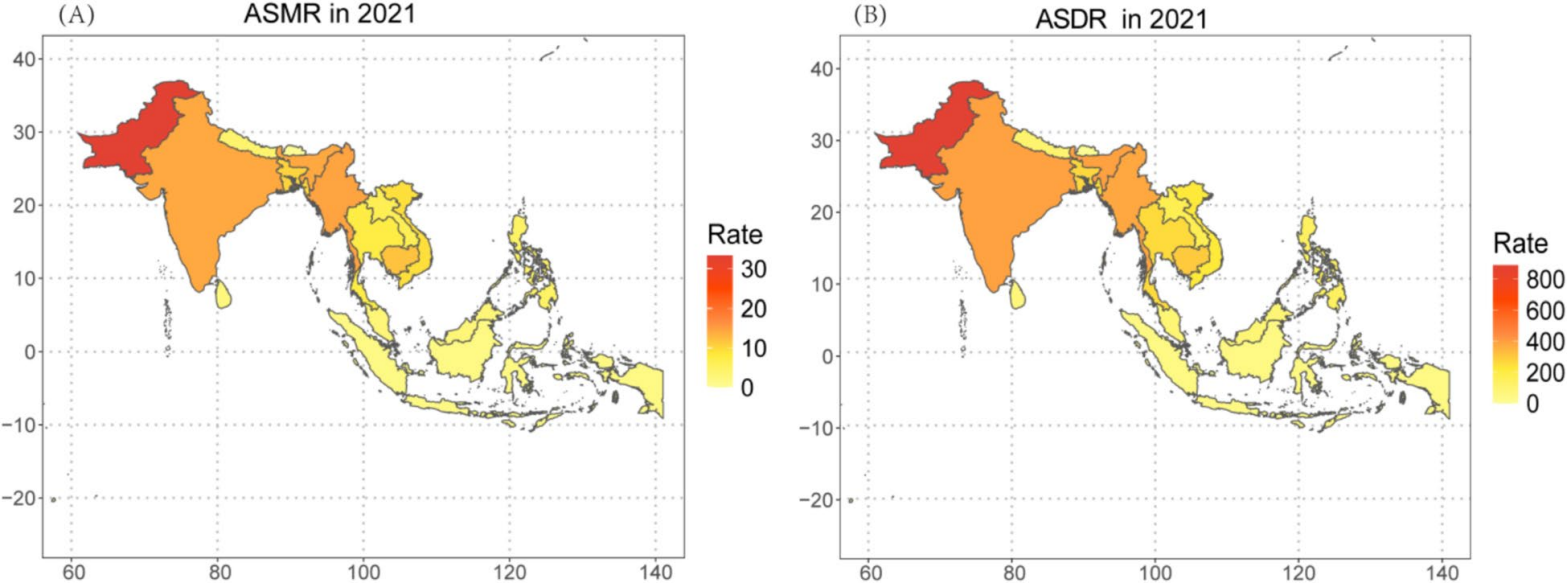
Maps of **A** age-standardized mortality rate (ASMR) and **B** age-standardized DALYs rate (ASDR) attributed to high temperature in South Asia and Southeast Asia in 2021

In 2021, South Asia experienced a higher burden of mortality and DALYs attributable to high temperature compared to Southeast Asia (Table S1). Approximately 209,537 deaths (95% UI: 141,705 to 282,078) and 6,777,284 DALYs (95% UI: 4,692,620 to 8,866,282) were recorded in South Asia (Table 1). In comparison, Southeast Asia reported 32,230 deaths (95% UI: 26,784 to 38,444) and 973,956 DALYs (95% UI: 840,628 to 1,128,262) attributable to high temperature (Table S1). Within South Asia, Pakistan had the highest ASMR and ASDR attributable to high temperature, followed by India, which accounted for the largest number of deaths and DALYs attributable to high temperature (Table S1; Figs. 2, 3). In contrast, Bhutan reported the lowest ASMR and an ASDR attributable to high temperature in South Asia (Table S1, Figs. 2, 3). In Southeast Asia, Myanmar exhibited the highest ASMR and ASDR attributable to high temperature (Table S1, Figs. 2, 3). Conversely, Mauritius recorded the lowest ASMR and ASDR attributable to high temperature (Table S1, Figs. 2, 3).

### Differences in disease burden attributed to high temperature across age and sex

**Fig. 4:**
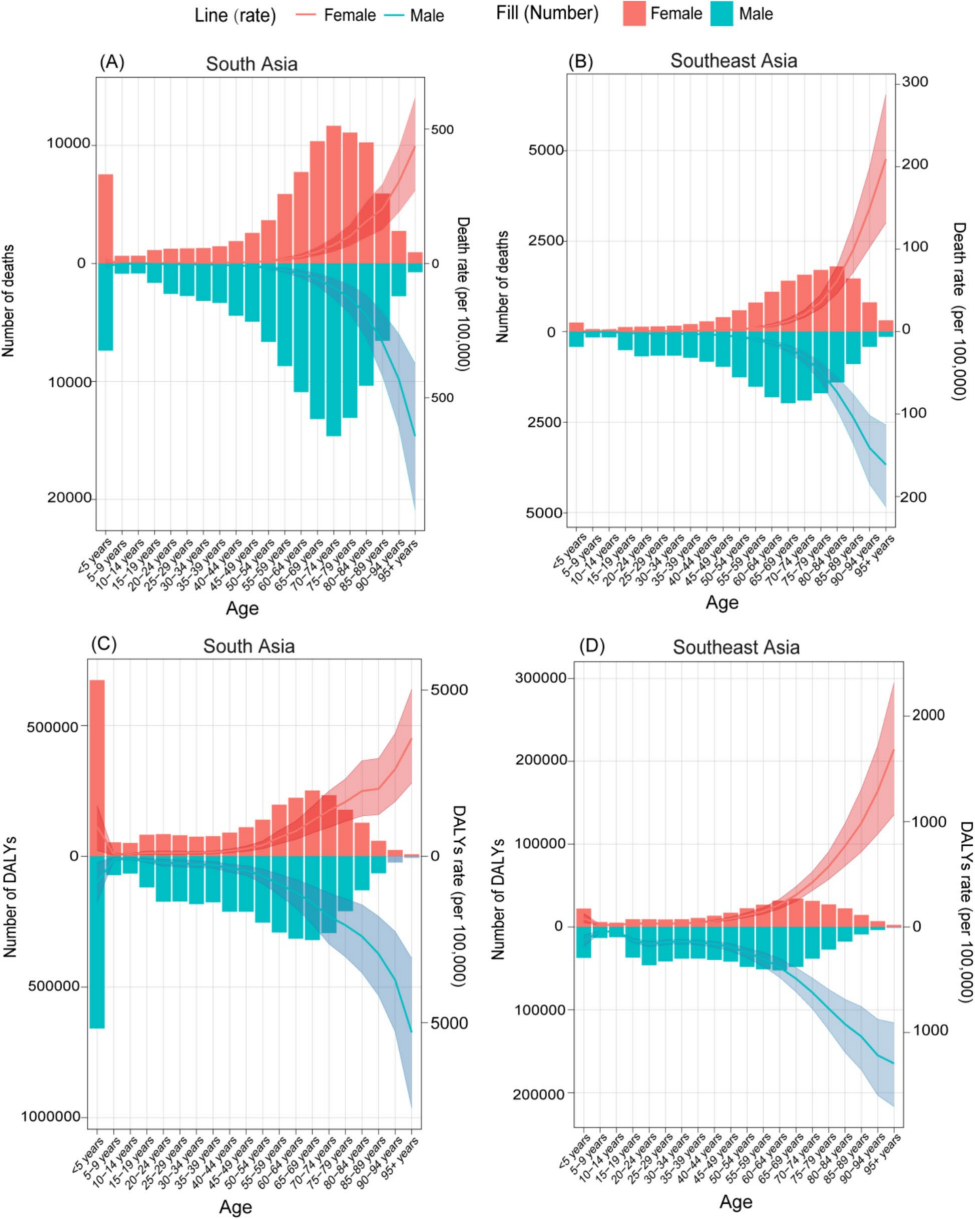
Numbers and rates of death attributed to high temperature in different age groups in **A** South Asia and **B** Southeast Asia in 2021

As age increases, rate of mortality and DALYs attributable to high temperature rise significantly from the 55–59-year age group onward in South Asia, with males experiencing a more pronounced increase compared to females. Notably, the 5 years and younger age group also exhibited higher rate of mortality and DALYs attributable to high temperature compared to the adjacent age groups (Fig. 4). In Southeast Asia, a similar pattern was observed (Fig. 4). In the older age groups, females experience higher rate of mortality and DALYs attributable to high temperature in Southeast Asia, whereas in South Asia, males have higher rate of mortality and DALYs in the same age groups (Fig. 4).

Moreover, the mortality rates attributable to high temperature across 14 countries in Southeast and South Asia showed a common trend of relatively low rate of mortality and DALYs for both males and females in younger age groups, but a sharp increase in those aged 55 years and above, with the steepest rises typically occurring in the 60–64-year and 70–74-year age groups (Figs. S1 and S2). In most countries, males exhibit higher rate of mortality and DALYs attributable to high temperature, particularly in older age groups, except for Malaysia and Philippines (Figs. S1 and S2).

### Trends of disease burden attributed to high temperature from 1990 to 2021

**Fig. 5:**
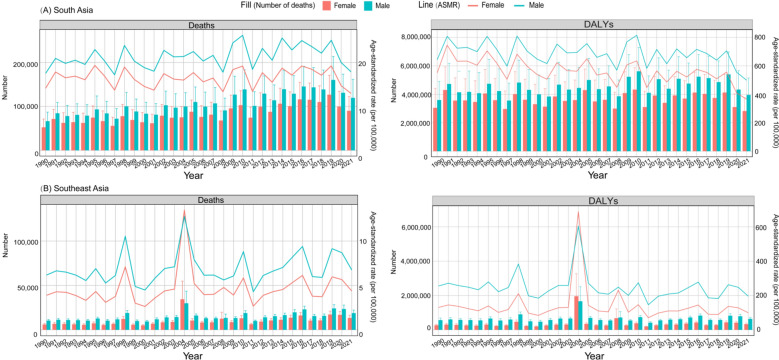
Trends of the age-standardized mortality rate (ASMR) and number of deaths attributed to high temperature in different countries of South Asia and Southeast Asia from 1990 to 2021

From 1990 to 2021, the ASMR attributed to high temperature in South Asia and Southeast Asia showed no significant change trend (Table S2). However, it is worth noting that from 1990 to 2019, ASMR of males attributed high temperature showed a significant increase in South Asia(APC: 0.71, 95% CI 0.17–8.13). From 1990 to 2021, the ASDR caused by high temperature in Southeast Asia did not show obvious change trend, while the ASDR caused by high temperature in South Asia decreased significantly (Table S3). Moreover, from 1990 to 2021, ASMR and number of deaths and DALYs attributed to high temperature were consistently higher for males than females in South Asia (Fig. 5).

In South Asia, Pakistan experienced significant increases in ASMR for both males (APC: 2.34, 95% CI 0.81–9.06) and females (APC: 2.76, 95% CI 1.21–7.13) until 2002, followed by a slight decline. In Bhutan, ASMR of male showed a notable upward trend (APC: 0.75, 95% CI 0.07–1.44) from 1990 to 2021, while female showed no significant change. In India, ASMR of male rose significantly (APC: 0.91, 95% CI 0.32–5.9) until 2019, followed by a slight decline, while ASMR of female exhibited no significant change. Trends of ASMR in other South Asian countries showed no significant changes. Moreover, from 1990 to 2021, the ASDR caused by high temperature in Bangladesh, Bhutan and Nepal has been significantly decreased, while the ASDR caused by high temperature in India and Pakistan began to decrease significantly after 2019 and 2002, respectively (Table S3). Between 1990 and 2021, the ASMR and ASDR for males were higher than for females in most countries in South Asia (Figs. S2 and S3).

In Southeast Asia, from 1990 to 2021, Indonesia, Vietnam, Mauritius, and Timor-Leste all showed substantial increases in ASMR for both males and females. In Seychelles, ASMR of female increased significantly (APC: 2.53, 95% CI 0.82–4.23), while ASMR of male showed no significant change. In Thailand, both male and female mortality rates declined over the period, with a particularly marked reduction in male mortality (Table S2). From 1990 to 2021, the ASDR caused by high temperature in Mauritius, Seychelles and female of Malaysia increased significantly, while the ASDR caused by high temperature in men in Maldives, Myanmar, Sri Lanka and female of Thailand decreased significantly. ASDR caused by high temperature in Philippines increased significantly from 1996, and ASDR caused by high temperature in male of Timor-Leste decreased first, and increased significantly from 2010 (Table S3).

### Leading causes of the disease burden attributable to high temperature

**Fig. 6:**
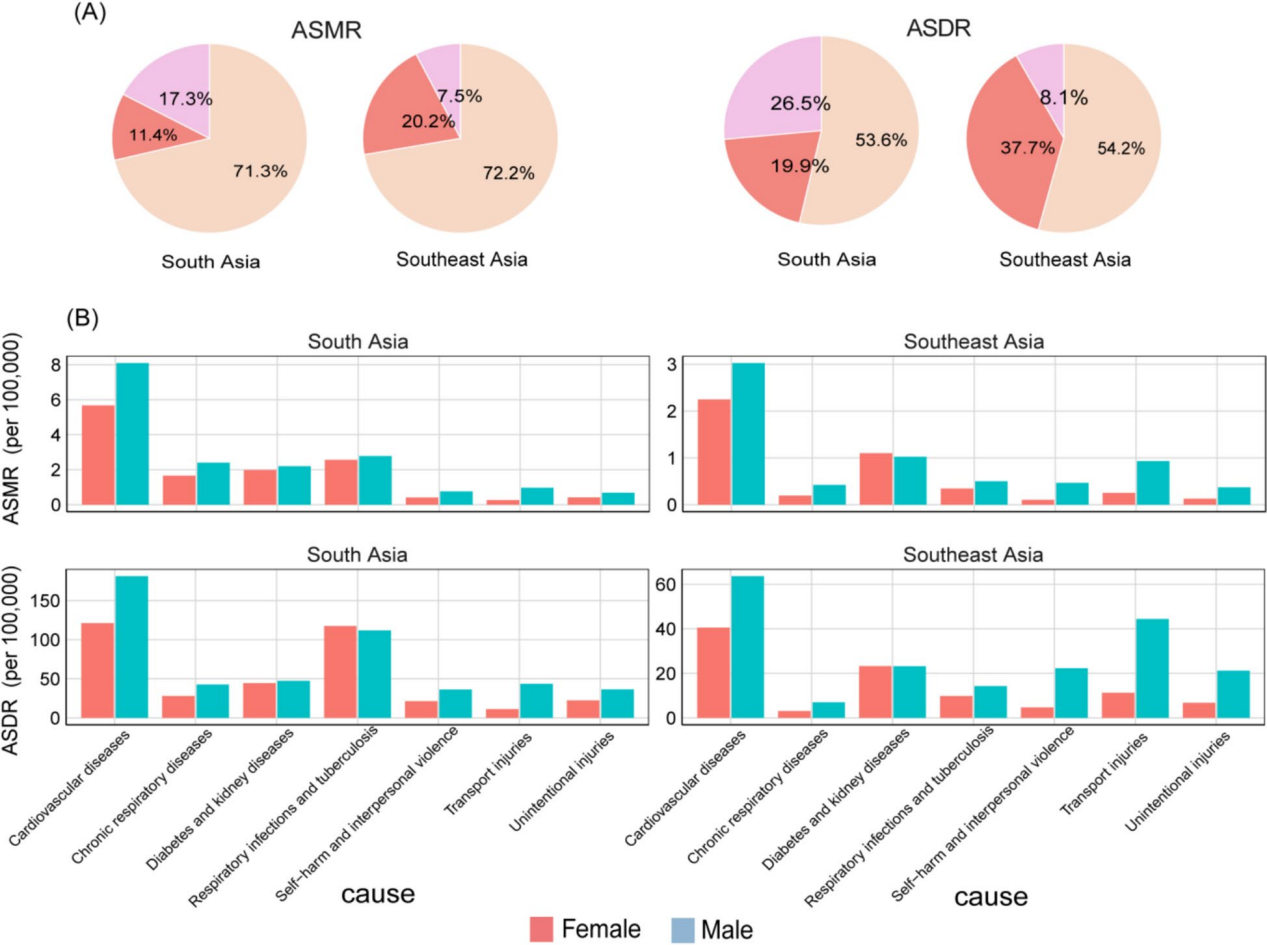
Causes of the age-standardized mortality rate (ASMR) attributed to high temperature in South Asia and Southeast Asia in 2021. **A** Level 1 and **B**, **C** Level 2 causes of ASMR attributed to high temperature in South Asia and Southeast Asia in 2021

The ASMR and ASDR attributed to high temperature in South and Southeast Asia was primarily driven by non-communicable diseases accounting for 70%-80% of the attributable disease burden (Fig. 6A, B). For each cause of GBD Level 2, cardiovascular disease was the predominant cause of high temperature-related ASMR and ASDR in both regions(Fig. 6C, D). Further analysis shows that among population under 5 years and over 55 years who bear higher diseases burden caused by high temperature, infectious diseases, communicable, maternal, neonatal, and nutritional diseases were the primarily causes of the ASMR and ASDR of children under 5 years, and non-communicable diseases were the primarily causes of the ASMR and ASDR of the elderly over 55 years (Figs. S4 and S5). As for cause of GBD Level 2, in South Asia, respiratory infections and tuberculosis were the primarily causes of the ASMR and ASDR of children under 5 years, while cardiovascular diseases were the primarily causes of the ASMR and ASDR of the elderly over 55 years. In contrast, in Southeast Asia, the main causes of ASMR and ASDR in children under 5 years and elderly people over 55 years were unintentional injuries (Figs. S4 and S5).

Notably, the main causes of high temperature-related ASMR and ASDR of most countries in South and Southeast Asia, were cardiovascular diseases, except Mauritius, Timor-Leste, Seychelles and Thailand (Figs. S6 and S7). The main causes of high temperature-related ASMR and ASDR was diabetes and kidney diseases in Mauritius, while respiratory infections and tuberculosis were the main causes of high temperature-related ASMR and ASDR in Timor-Leste. Thailand and Seychelles exhibited gender-specific patterns in the main causes of high temperature-related ASMR and ASDR. In Thailand, the main causes of high temperature-related ASMR and ASDR for females were cardiovascular disease, whereas for males, it was transport injuries. In Seychelles, the main causes of high temperature-related ASMR and ASDR for females were diabetes and kidney diseases, while for males, it was respiratory infections and tuberculosis (Figs. S6 and S7).

### Age-period-cohort effects on disease burden attributed to high-temperature

**Fig. 7:**
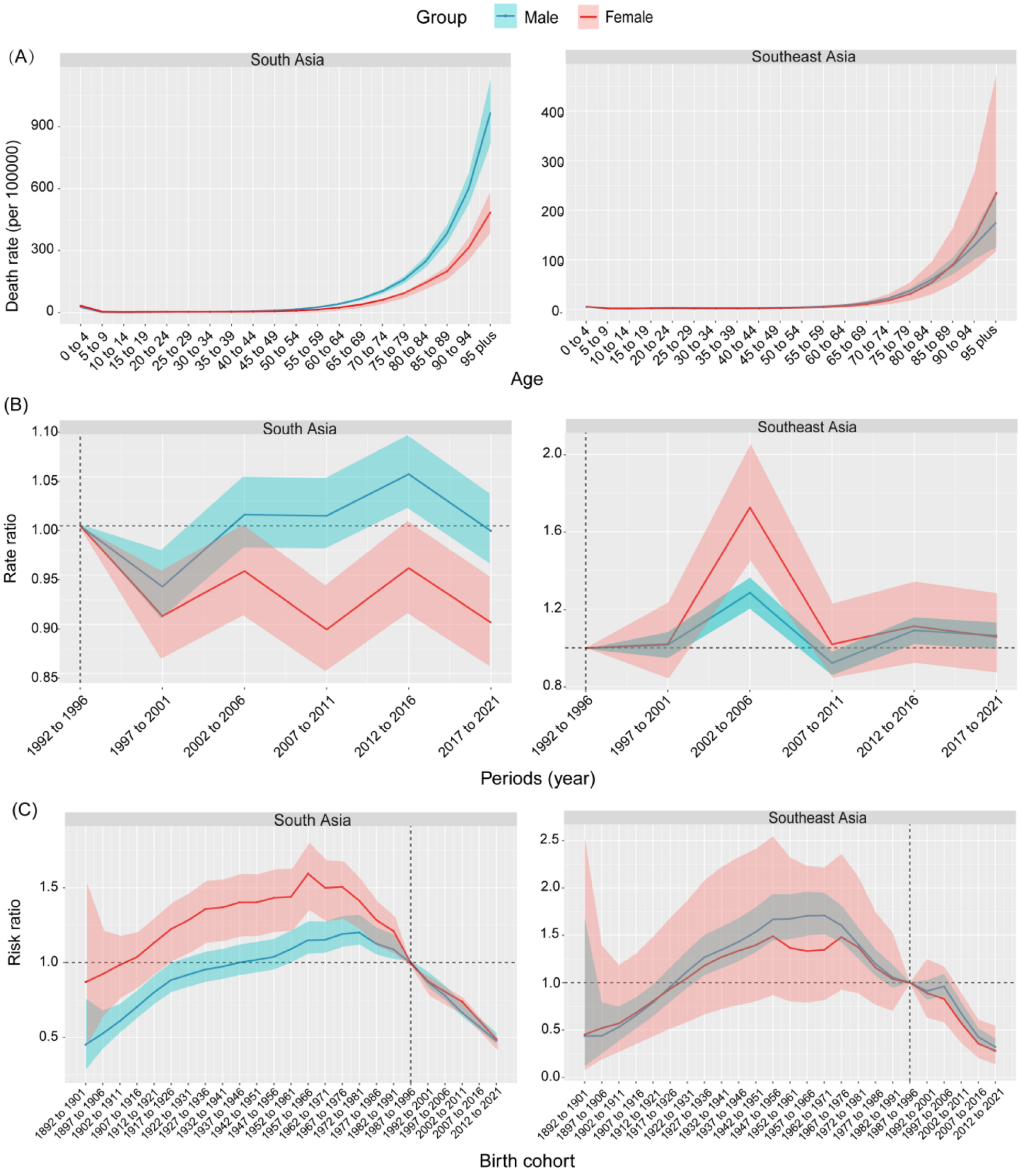
Age, period, and cohort effects on the disease burden attributed to high temperature in South Asia and Southeast Asia. **A**–**C** Age, period, and cohort effects, respectively

In general, the age effect shows a significant increase in rate of mortality and DALYs with advancing age in both Southeast and South Asia, with peak rates observed in the elderly (Figs. 7A and S8A). The period effect showed relative risk fluctuations of death and DALYs over time, with more pronounced variations observed in South Asia (Figs. 7B and S8B). Specifically, in the 2002–2006 period, both males and females in Southeast Asia experienced higher relative risks in death and DALYs (Figs. 7B and S8B). Regarding birth cohorts, the risk initially increased and then decreased in both regions, with a decline in risk observed in more recent cohorts (Fig. 7C). The mortality rate for females born before 1987–1996 In South Asia was generally higher than that for males (Fig. 7C). It is worth noting that a similar trend has been found in DALYs (Fig. S8C).

### Decomposition analysis of disease burden attributed to high-temperature

**Fig. 8:**
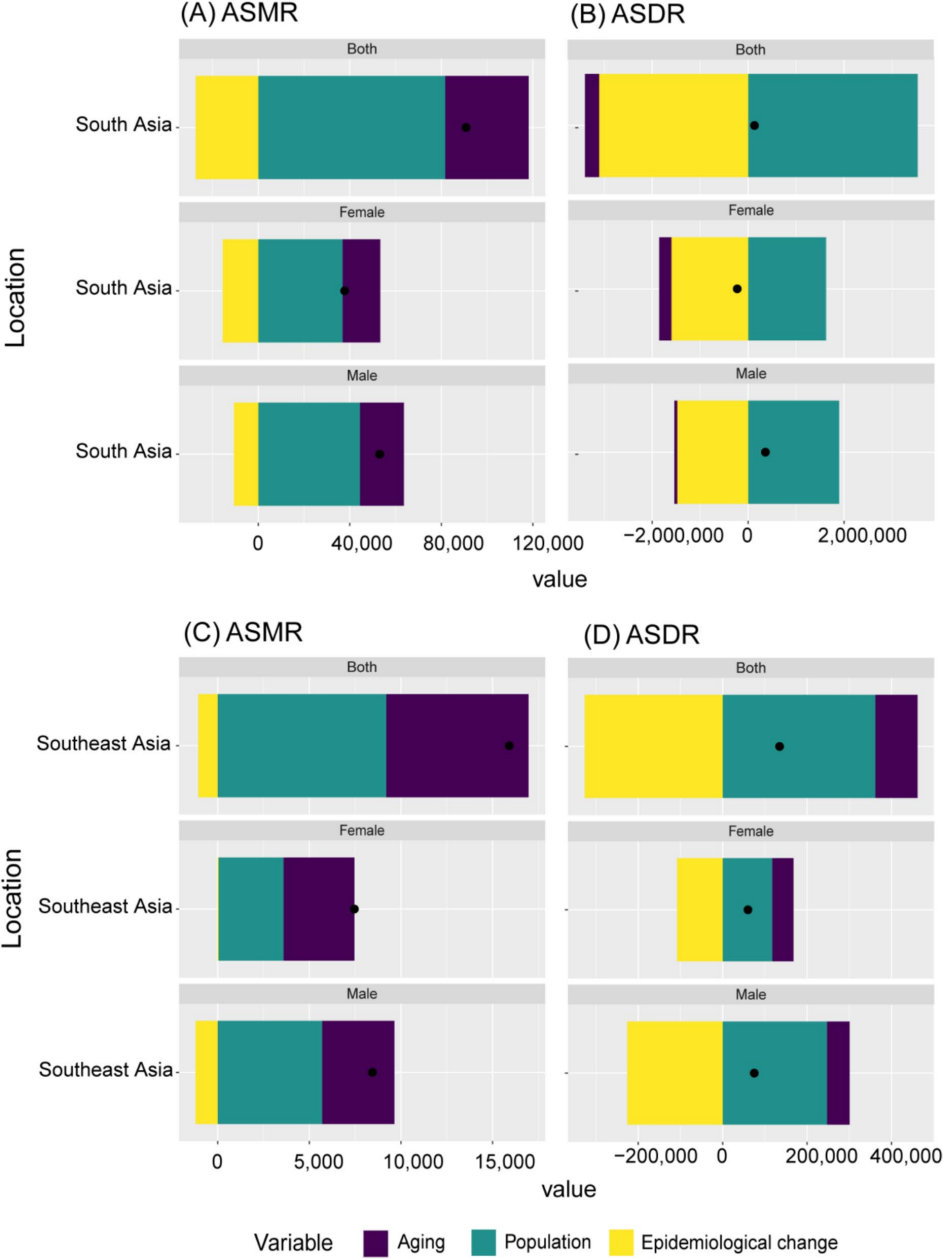
Decomposition analysis of the **A** age-standardized mortality rate (ASMR) attributed to high temperature in Southeast Asia and South Asia and **B** age-standardized DALYs rate (ASDR) attributed to high temperature in Southeast Asia and South Asia

The ASMR in South Asia has increased due to population growth and aging, while epidemiological changes have inhibited its growth. However, ASDR in South Asia presents a different pattern: population growth leads to an increase in ASDR, while aging and epidemiological changes play a role in reducing it (Fig. 8A, B). In contrast, the increase in ASMR and ASDR in Southeast Asia was primarily driven by population growth and aging, while improvements in epidemiological changes contributed to a reduction in ASMR and ASDR (Fig. 8C, D). In South Asia, epidemiological changes and aging reduced the ASMR in Pakistan, while population growth increased the ASMR (Fig. S9). In contrast, population growth and aging increased the ASMR in other South Asian countries, while epidemiological changes reduced it (Fig. S9). Epidemiological changes and population aging reduced the ASDR in South Asian countries, while population growth led to an increase in ASDR (Fig. S9).

### Prediction of disease burden attributed to high-temperature to 2045

**Fig. 9:**
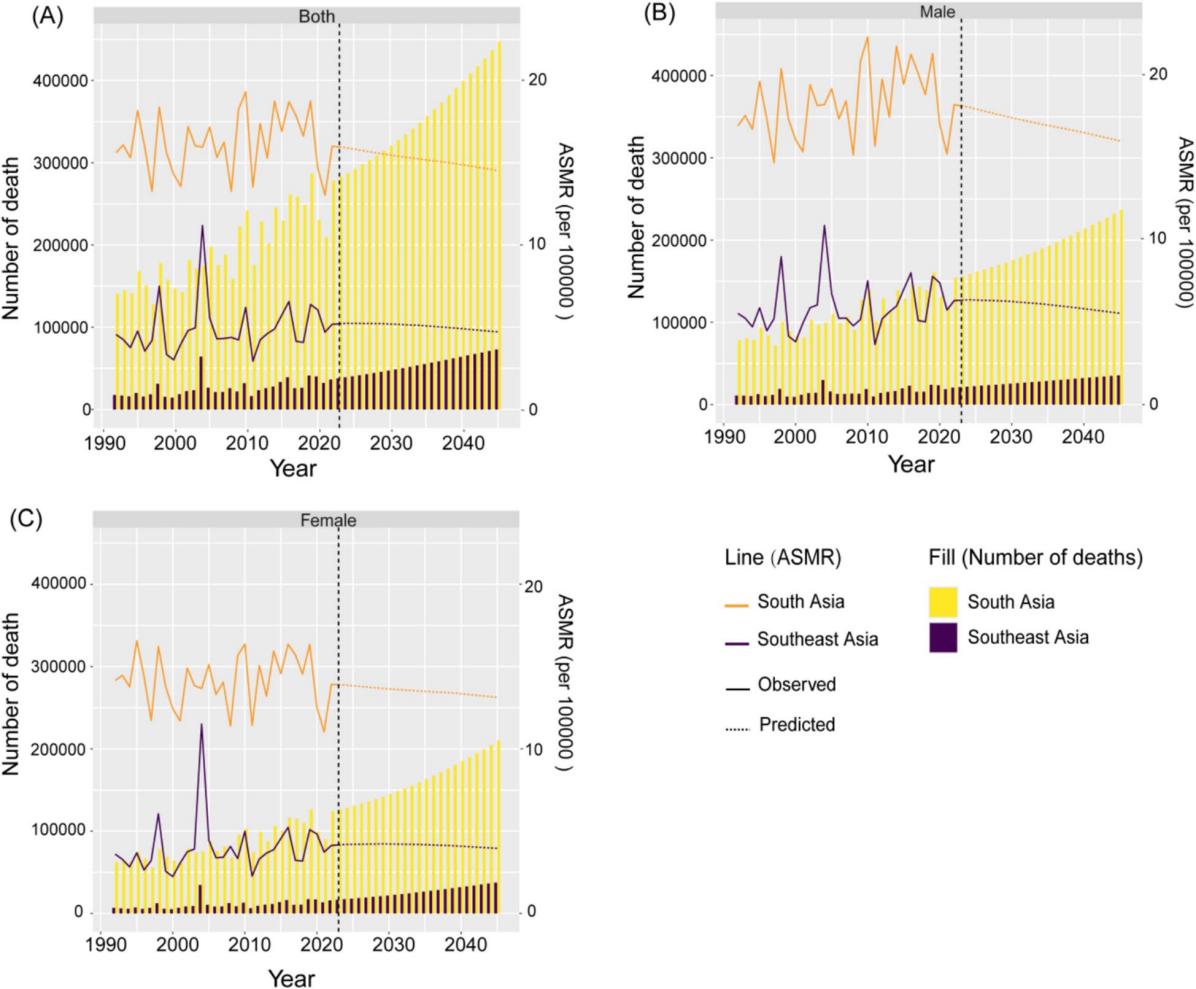
Projected number of deaths and age-standardized mortality rate (ASMR) attributed to high temperature in South and Southeast Asia from 2021 to 2045: **A** both Sexes, **B** male, and **C** female

A projected increase in the total number of deaths due to high temperature in South and Southeast Asia from 2021 to 2045 was anticipated. However, the ASMR due to high temperature in both South and Southeast Asia is expected to decrease over this period, with a more pronounced decline in South Asian males compared to females (Fig. 9). Moreover, from 2021 to 2045, the ASMR and the number of deaths caused by high temperature in South Asia are projected to be much higher than those in Southeast Asia (Fig. 9). For different countries in South and Southeast Asia, the high temperature-related ASMR in Timor-Leste, Mauritius, Sri Lanka and Bangladesh are expected to increase, while the high temperature-related ASMR in Malaysia, Lao People's Democratic Republic, Philippines, and Viet-Nam are projected to increase initially followed by subsequent decrease. In addition, the high temperature-related ASMR in Myanmar, India, Thailand, Bhutan, Cambodia, Maldives, Seychelles and Nepal were expected to decline in the future. In the remaining countries, ASMR related to high temperature is not expected to change in the future (Fig. S10).

## Discussion

This study focused on the disease burden caused by high temperature in South Asia and Southeast Asia, where children under 5 years and elderly people over 55 years bear the heaviest disease burden, and the disease burden caused by high temperature was higher in male than in female. On the whole, non-communicable diseases, especially cardiovascular diseases, are the main causes of high temperature related burden in this area. However, there are differences in gender distribution and main causes in high temperature related burden of different countries. Although the death rate related to high temperature in South Asia and Southeast Asia would decrease in the future, the death toll may still increase.

Specifically, this study showed that the disease burden attributed to high temperature is disproportionately concentrated in South Asia, accounting for 46–51% of global deaths and DALYs related to high temperatures. This is mainly due to the dense populations, limited adaptive capacity and high baseline temperature in South Asia [29]. Notably, the surge in high temperature-related disease burden across Southeast Asia in 2004 may be linked to the Indian Ocean tsunami [30]. This catastrophic event displaced large populations and degraded public health infrastructure, exacerbating disease transmission [31]. Consequently, the capacity of affected communities to cope with other environmental risks diminished, demonstrating synergistic interactions among environmental risk factors [32]. In 2021, South Asia experienced a higher mortality and DALYs attributable to high temperature compared to Southeast Asia. Among them, Pakistan exhibited the highest ASMR attributed to high temperature in South Asia, while Myanmar led in Southeast Asia. These patterns correlate with regional climatic extremes, accelerating urbanization rates and healthcare access disparities. In addition, Pakistan and India, further buttress studies that stipulated that low and middle-income countries bear the brunt of climate-related health risks, mainly attributed to limited adaptive capacity and healthcare infrastructure [33, 34].

Moreover, the gender disparity of the mortality caused by high temperature in South Asia and Southeast Asia was evidently pronounced, with males experiencing significantly higher mortality rates than females. This observed gender gap aligns is due to the fact that males bear an increased occupational and behavioral exposure to high temperature, especially in South Asia and Southeast Asia, where they are more likely to engage in outdoor labor [35, 36]. Hence, the shift from communicable, maternal, neonatal, and nutritional diseases to injuries as the leading cause of heat-related mortality among males in Southeast Asia underscores the need for occupational safety measures and heat adaptation strategies tailored to high-risk industries. It is worth noting that the age-period-cohort analysis found that the risk for older female cohorts in South Asia is consistently higher than that for males, a pattern potentially attributable to more traditional social structures in South Asia that disproportionately limit older females' access to resources and healthcare [37].

We found relatively higher mortality rate and DALYs attributed to high temperature in children under 5 years in South Asia and Southeast Asia, and it was primarily cause driven by communicable, maternal, neonatal, and nutritional diseases. This may be due to the fact that high temperature can facilitate transmission of infectious diseases such as diarrhea and exacerbate the incidence of vector-borne diseases, such as malaria and dengue. In addition, high temperature increase the risk of complications during pregnancy and childbirth, contributing to preterm births and low birth weight [38]. Furthermore, extreme heat can suppress appetite [39], leading to malnutrition in children. As for elderly over 55 years in Southeast Asia and South Asia, rates and numbers of death attributed to high temperature increased significantly with age, which were mainly caused by cardiovascular diseases. This can be attributed to the reduced efficiency of the cardiovascular system in response to environmental temperature as people age, making older adults more susceptible to heat stress and exacerbating pre-existing cardiac conditions [40, 41]. Chronic diseases prevalent in the elderly population, such as hypertension, diabetes, and obesity, further intensify cardiovascular strain during extreme heat events.

From the perspective of the whole population, in South Asia and Southeast Asia, mortality attributed to high temperature exhibited a predominantly etiological pattern with non-communicable diseases, especially cardiovascular diseases as the leading cause. However, notable heterogeneity exists in the pattern of secondary causes vary regionally, with communicable, maternal, neonatal, and nutritional diseases assuming significant predominance prominent in South Asia, while injuries assume more significance in Southeast Asia. Furthermore, gender-specific patterns were observed in countries, such as Thailand and Seychelles, highlighting the complex interplay between mortality attributed to high temperature and regional health determinants.

Furthermore, we project a decline in ASMR from 2021 to 2045 most countries in South Asia and Southeast Asia, but an increase in absolute deaths, in which aging populations and population growth offset improvements in healthcare and climate adaptation. However, countries such as Timor-Leste, Mauritius, Sri Lanka and Bangladesh are expected to experience increasing ASMR, highlighting the need for targeted interventions. In addition, there are limitations in this study. The inherent biases in GBD-modeled data (e.g., outdated correction factors) and limitations in predictive assumptions (e.g., static vulnerability), potentially skewing disease burden estimates.

These findings highlight several policy implications. First, we believe there's an urgency to strengthen healthcare systems focusing on high temperature in South Asia and Southeast Asia, including providing adequate maternal and child health services, expanding vaccination and enhancing chronic disease management, to address the persistent high burden caused by high temperature among children and older adults. Second, occupational safety measures are essential to reduce high temperature related injuries among working-age male. Third, early warning systems, shelters and urban heat mitigation measures can be prioritized as climate adaptation strategies protect vulnerable populations.

## Conclusions

The disease burden caused by high temperature is disproportionately concentrated in South Asia and Southeast Asia, which presents a critical public health challenge that requires urgent intervention. In particular, children under 5 years and the elderly over 55 years bear the heaviest burden of disease. On the whole, males in South Asia and Southeast Asia bear a higher disease burden attributable to high temperatures than females. Non-communicable diseases, particularly cardiovascular diseases, were the leading cause of the disease burden attributable to high temperature in South Asia and Southeast Asia. However, it is worth noting that the disease burden caused by high temperature in different countries in South Asia and Southeast Asia has different gender patterns and leading cause. In South Asia and Southeast Asia, aging and population growth offset improvements in ASMR caused by epidemiological change. More importantly, there's a projected increase in number of deaths despite declining rates in South Asia and Southeast Asia in the future. These findings emphasize the urgent need to reduce the diseases burden caused by high temperature in South Asia and Southeast Asia, especially to take intervention measures for different regions and vulnerable groups of children under 5 years and the elderly over 55 years. In addition, this study did not account for potential interactions between high temperature and other climate-related hazards, which needs to be further explored in future research.

## Supplementary Information

Additional file 1.

## Data Availability

Data used in this study were obtained from the Global Health Data Exchange Global Burden of Disease Results Tool (https://ghdx.healthdata.org/gbd-results-tool).

## Abbreviations

DALYs: Disability-adjusted life years;
ASMR: The age-standardized mortality rate;
ASDR: The age-standardized DALYs rate;
ASR: The age-standardized rate;
GBD: Global disease burden;
APC: Annual percent change;
AAPC: Average annual percent change;
UI: Uncertainty intervals
CI: Confidence interval
APCM: Age-Period-Cohort model
BAPCM: Bayesian Age-Period-Cohort Model
