# Supplementary material for: Disease burden attributable to high temperature between 1990 and 2021 in South Asia and Southeast Asia, with projections to 2045
Source: Trop Med Health. 2025 Oct 30;53:146. doi: 10.1186/s41182-025-00823-4 (PMC12573994; doi:10.1186/s41182-025-00823-4)
Supplement: Supplementary file 1 — Additional file 1. [file 41182_2025_823_MOESM1_ESM.docx]

Disease Burden Attributable to High temperature between 1990 and 2021 in South Asia and Southeast Asia, with Projections to 2045

Jingfang Cai ^1†*^, Ahiafor Maxwell ^1†^, Boda Zhou ^1*^

**Affiliations**

^1^Beijing Tsinghua Changgung Hospital, School of Clinical Medicine, Tsinghua University, Beijing 102218, China.

†Jingfang Cai and Ahiafor Maxwell contributed equally to this work and share first authorship.

**Correspondence:**

Jingfang Cai, Ph.D.

Beijing Tsinghua Changgung Hospital, School of Clinical Medicine, Tsinghua University

Address: 168 Litang Road, Changping District, Beijing 102218, China

Phone: 086-010-56119519, fax: 086-010-56118972

E-mail: caijf@mail.tsinghua.edu.cn

Or

Boda Zhou, M.D.

Beijing Tsinghua Changgung Hospital, School of Clinical Medicine, Tsinghua University

Address: 168 Litang Road, Changping District, Beijing 102218, China

Phone: 086-010-56119519, fax: 086-010-56118972

E-mail: zhouboda@tsinghua.edu.cn

**Supplementary materials:**

- Appendix 1.Supplementary methods
- Appendix 2.Table S1-S3
- Appendix 3.Figure Figure S1~S10

Appendix 1.Supplementary methods

1. **Estimated disease burden due to high temperatures**

GBD study adopted internationally recognized unified standardized data processing methods to systematically estimate cause-specific deaths and disability-adjusted life years (DALY) [1]. The methods for estimating the disease burden attributable to high temperature are similar to that in other risk factors. Briefly, it includes the following steps: (1) Data collection: the temperature estimates were obtained from the grid reanalysis dataset ERA5 generated by the European Centre for Medium-Range Weather Forecasts and then calculated daily averages of temperatures for use in burden estimation. The ERA5 reanalysis data set, a product of the European Centre for Medium-Range Weather Forecasts that provides a high spatial resolution of 0.25℃× 0.25℃ from 1979 up until the present. Individual mortality data were obtained from the GBD cause of death (CoD) database for vital registration data sources .Finally, 12 disease causes (Level 3) were included as the outcome related to high temperature (Figure S1), but this study used Level 1 and Level 2 disease causes for analysis. (2) Exposure-response modelling: the theoretical minimum risk exposure level (TMREL) for temperature in each given location and year, defined as the the temperatures with the lowest mortality risk. High temperature exposure referred to exposure to temperatures greater than TMREL. Temperature zones were defined based on mean annual temperature. A robust meta-regression framework implemented through the MR-BRT tools including MR-Bayesian, regularized, trimmed, was used to estimate cause-specific mortality according to average daily temperature and temperature zone. (3) Calculation of disease burden indicators: death and DALY due to the specific cause were estimated were estimated according to internationally recognized standard methods. Detailed methods has been introduced in previous studies[1,2,3,4,5].

1. **Joinpoint Regression**

**Model Specification**

Joinpoint regression typically employs two primary model formulations. When the dependent variable follows a normal (or approximately normal) distribution with sufficiently large sample sizes (typically n > 100), the linear model (y = βx) is generally applied. Conversely, for exponentially distributed or Poisson-distributed dependent variables, the logarithmic linear model (ln(y) = βx) is more appropriate. In the above formula, x is an independent variable, representing time; And y is the dependent variable representing the diseases data related to high temperature (such as mortality and DALY).

**Grid Search Methodology**​

The grid search method (GSM) constitutes the foundational optimization algorithm in Joinpoint regression. Operationally, GSM exhaustively evaluates potential segmentation points while deriving corresponding sum of squared errors (SSE) and mean squared error (MSE) metrics for each piecewise function configuration. Nodal positions yielding minimal MSE values are prioritized as optimal joinpoints, with regression coefficients subsequently estimated through iterative optimization of selected segments .

**Model Optimization**​

Monte Carlo permutation testing serves as the primary model optimization technique. During modeling, the permissible range for joinpoint count (k) is bounded between minimum (MIN) and maximum (MAX) values. Sequential permutation testing evaluates competing hypotheses by contrasting a null model (H₀: k = MIN) against an alternative model (H₁: k = MAX). The procedure iteratively adjusts k: rejecting H₀ triggers incremental augmentation (k+1), while failure to reject H₀ prompts decremental reduction (k–1). This iteration continues until MIN = MAX, indicating the optimal joinpoint count, finalizing model selection .

**Index Calculation**​

1. **APC is derived as:**

APC=($\text{e}^{\text{β}\text{1}}$−1)×100

*e*: Base of natural logarithm;

β1: Regression coefficient from log-linear joinpoint model (ln(y) = β₀ + β₁x)

Confidence intervals of APC are calculated as:

APC_L(α)_ =100×($\text{e}^{\text{β}\text{1-}\text{s}\text{t}_{\text{d}}^{\text{-}\text{1}}\text{(1-}\text{α}\text{/2) }}\text{-}\text{1)}$

APC_U(α)_ =100×($\text{e}^{\text{β}\text{1-}\text{s}\text{t}_{\text{d}}^{\text{-}\text{1}}\text{(1-}\text{α}\text{/2) }}\text{-}\text{1)}$

β1: Regression coefficient from log-linear joinpoint model

s:standard error of β1;

d: degrees of freedom

1. **AAPC is derived as:**

AAPC=($\text{e}^{\sum{\text{w}_{\text{i}}\text{β}}_{\text{i}}\text{/}\sum\text{w}_{\text{i}}}\text{-}\text{1)×100}$

w_i_: Number of years in each trend segment;

β_i_: Segment-specific regression coefficients;

The ratio $\sum{\text{w}_{\text{i}}\text{β}}_{\text{i}}\text{/}\sum\text{w}_{\text{i}}$ computes the duration-weighted average of all β coefficients

Confidence intervals of APC are calculated as:

AAPC_L(α)_ = {exp[$\ln\text{(}\frac{\text{AAPC}}{\text{100}}\text{+1)-}\text{Z}_{\text{1-}\frac{\text{α}}{\text{2}}}\sqrt{\sum{\text{ω}_{\text{i}}^{\text{2}}\text{σ}}_{\text{i}}^{\text{2}}}\text{]-1\}}$

AAPC_U(α)_ = {exp[$\ln\text{(}\frac{\text{AAPC}}{\text{100}}\text{+1)-}\text{Z}_{\text{1-}\frac{\text{α}}{\text{2}}}\sqrt{\sum{\text{w}_{\text{i}}^{\text{2}}\text{σ}}_{\text{i}}^{\text{2}}}\text{]-1\}}$

w_i_: the width of each segment function interval;

$\text{σ}_{\text{i}}$: the variance of βi;

βi: Segment-specific regression coefficients;

Z: corresponding value of the α percentile in the normal distribution

### Decomposition Analysis

The decomposition analysis of deaths and DALYs in each region is obtained by the following formula:

D_ay, py, ry_ = $\sum_{i=1}^{4} ($a _i, y_ × p _y_ × r _i, y_)

D_ay, py, ry_ : Deaths or DALYs based on the age structure, population, and rate of deaths or DALYs in specific year y;

a _i, y_: The population proportion for the specific age category i of the 4 age categories in specific year y;

p _y_ : The total population in specific year y;

r _i, y_ : The rate of deaths or DALYs of specific age category i in specific year y.

Reference

[1] GBD 2021 Risk Factors Collaborators. **Global burden and strength of evidence for 88 risk factors in 204 countries and 811 subnational locations**, 1990-2021: a systematic analysis for the Global Burden of Disease Study 2021. *Lancet*. 2024, **403**(10440):2162-2203.

[2] Qu C, Chen Y, Liu C, Hu Z, Zhang J, Yan L, Zhang H, Liu Y, Liu W, Cheng Q, Luo P, Liu Z. **Burden of Stroke Attributable to Nonoptimal Temperature in 204 Countries and Territories: A Population-Based Study, 1990-2019**. *Neurology*. 2024, **102**(9):e209299.

[3] GBD 2019 Diseases and Injuries Collaborators. **Global burden of 369 diseases and injuries in 204 countries and territories, 1990-2019: a systematic analysis for the Global Burden of Disease Study 2019**. *Lancet*. 2020, **396**(10258):1204-1222.

[4] Al-Kindi S, Motairek I, Khraishah H, Rajagopalan S. **Cardiovascular disease burden attributable to non-optimal temperature: analysis of the 1990-2019 global burden of disease**. European Journal of Preventive Cardiology. 2023, **30**(15):1623-1631.

[5] Song J, Pan R, Yi W, Wei Q, Qin W, Song S, Tang C, He Y, Liu X, Cheng J, Su H.. **Ambient high temperature exposure and global disease burden during 1990-2019: an analysis of the Global Burden of Disease Study 2019**. *Science Total Environment*. 2021, **787**:147540.

- Appendix 2.Table S1-S3

Table S1. The number and the age-standardized rate (ASR) of deaths and DALYs caused by high temperatures in South Asia and Southeast Asia in 2021, as well as the trends in age-standardized rates of deaths and DALYs from 1990 to 2021.

Table S2. The annual percent change (APC) and average annual percentage change (AAPC) of the age-standardized mortality rate (ASMR) attributed to high temperature in South Asia and Southeast Asia from 1990 to 2021 based on the joinpoint regression analysis model.

Table S3. The annual percent change (APC) and average annual percentage change (AAPC) of the age-standardized DALYs rate (ASDR) attributed to high temperature in South Asia and Southeast Asia from 1990 to 2021 based on the joinpoint regression analysis model.

Table S1. The number and the age-standardized rate (ASR) of deaths and DALYs caused by high temperatures in South Asia and Southeast Asia in 2021, as well as the trends in age-standardized rates of deaths and DALYs from 1990 to 2021.

| **Location** | **Sex** | **Deaths in 2021 (95% UI)** | | **DALYs in 2021(95% UI)** | |
| --- | --- | --- | --- | --- | --- |
|  |  | **Count number** | **ASR (per100,000,)** | **Count number** | **ASR (per100,000,)** |
| **South Asia** | | | | | |
| South Asia | Female | 90061 (59612,122391) | 12.96 (8.51,17.8) | 2822063 (1970115,3763712) | 365.79 (250.73,489.48) |
|  | Male | 119476 (79577,161865) | 17.89 (11.66,24.74) | 3955221 (2708619,5148711) | 498.86 (342.43,654.25) |
|  | Both | 209537 (141705,282078) | 15.31 (10.14,20.99) | 6777284 (4692620,8866282) | 431.29 (297.52,567.63) |
| Bangladesh | Female | 5242 (2836,7972) | 9.06 (4.74,13.91) | 151945 (97229,216432) | 225.22 (141.38,323.81) |
|  | Male | 7560 (4549,10928) | 11.73 (6.93,17.01) | 226175 (149647,318879) | 311.1 (202.93,440.67) |
|  | Both | 12802 (7526,18856) | 10.41 (6.06,15.37) | 378119 (252959,532467) | 267.69 (176.19,378.89) |
| Bhutan | Female | 1 (1,1) | 0.14 (0.01,0.35) | 11 (1,24) | 3.47 (0.39,8.18) |
|  | Male | 1 (1,1) | 0.17 (0.01,0.43) | 14 (1,32) | 4.24 (0.44,9.89) |
|  | Both | 2 (1,3) | 0.15 (0.02,0.37) | 25 (3,55) | 3.87 (0.48,8.77) |
| India | Female | 66758 (43986,90730) | 11.85 (7.72,16.18) | 1966981 (1339001,2653000) | 326.12 (219.4,440.35) |
|  | Male | 89179 (58509,119903) | 16.9 (11.1,23.11) | 2857881 (1965950,3740585) | 464.22 (317.06,613.79) |
|  | Both | 155937 (103632,211091) | 14.23 (9.25,19.49) | 4824862 (3337535,6321893) | 393.95 (270.64,520.05) |
| Nepal | Female | 384 (109,746) | 3.5 (0.95,6.95) | 11625 (4080,20377) | 87.95 (30.01,157.02) |
|  | Male | 468 (153,843) | 4.66 (1.4,8.62) | 15074 (5747,25482) | 121.46 (42.24,205.8) |
|  | Both | 852 (262,1556) | 4.04 (1.2,7.61) | 26699 (10355,45831) | 103.5 (35.93,178.97) |
| Pakistan | Female | 17676 (11063,26214) | 31.18 (19.64,47.15) | 691502 (419500,978841) | 821.29 (509.6,1204.8) |
|  | Male | 22269 (14146,31710) | 35.32 (21.59,51.85) | 856078 (525811,1179114) | 952.44 (616.11,1333.45) |
|  | Both | 39944 (25997,56001) | 33.31 (20.98,47.46) | 1547580 (964100,2158885) | 888.09 (570.6,1245.36) |
| **Southeast Asia** | | | | | |
| Southeast Asia | Female | 13408 (10863,16450) | 4.37 (3.49,5.37) | 332269 (279527,397011) | 99.37 (83.03,119.28) |
|  | Male | 18822 (15824,22215) | 6.74 (5.54,8.1) | 641687 (555368,731596) | 195.9 (169.35,225.64) |
|  | Both | 32230 (26784,38444) | 5.51 (4.51,6.68) | 973956 (840628,1128262) | 147.03 (126.57,171.13) |
| Cambodia | Female | 616 (475,763) | 10.01 (7.66,12.12) | 16191 (12211,20965) | 222.5 (171.3,282.55) |
|  | Male | 704 (548,874) | 14.48 (11.53,17.28) | 25422 (18977,34029) | 387.17 (298.41,488.75) |
|  | Both | 1320 (1037,1643) | 11.94 (9.35,14.4) | 41612 (31481,54660) | 299.3 (230.89,379.98) |
| Indonesia | Female | 1512 (1140,1930) | 1.45 (1.12,1.85) | 43644 (32805,55661) | 35.35 (26.98,44.89) |
|  | Male | 2172 (1684,2801) | 2.01 (1.58,2.56) | 82031 (61668,109875) | 62.78 (48.05,82.29) |
|  | Both | 3684 (2904,4610) | 1.73 (1.38,2.12) | 125675 (99029,156583) | 49.24 (39.37,60.86) |
| Lao People's Democratic Republic | Female | 122 (76,180) | 5.53 (3.36,8.16) | 4201 (2579,6167) | 144.12 (90.68,212) |
|  | Male | 157 (101,226) | 7.23 (4.47,10.66) | 6253 (4184,9009) | 207.16 (136.14,296.45) |
|  | Both | 279 (179,407) | 6.34 (3.91,9.29) | 10454 (6843,14981) | 175.27 (112.78,254.61) |
| Malaysia | Female | 283 (244,321) | 2.26 (1.91,2.61) | 7423 (6544,8477) | 51.85 (45.63,58.94) |
|  | Male | 542 (471,618) | 3.68 (3.25,4.17) | 19956 (15634,23433) | 121.09 (98.29,141.02) |
|  | Both | 825 (734,935) | 2.98 (2.63,3.37) | 27378 (22486,31724) | 87.47 (73.69,100.38) |
| Maldives | Female | 2 (1,2) | 1.34 (0.95,1.8) | 42 (32,55) | 27.56 (20.6,35.86) |
|  | Male | 4 (3,6) | 2.09 (1.58,2.71) | 164 (100,220) | 58.27 (39.69,77.98) |
|  | Both | 6 (5,8) | 1.77 (1.34,2.31) | 207 (134,276) | 45.98 (32.83,61.09) |
| Mauritius | Female | 3 (1,7) | 0.31 (0,0.73) | 65 (1,150) | 7.16 (0.09,16.24) |
|  | Male | 4 (1,8) | 0.49 (0,1.09) | 105 (1,222) | 13.32 (0.06,27.9) |
|  | Both | 8 (1,16) | 0.39 (0,0.9) | 168 (2,373) | 10.14 (0.07,21.79) |
| Myanmar | Female | 2974 (2208,3975) | 12.11 (8.99,16.18) | 40109 (32297,48583) | 291.91 (216.94,383.55) |
|  | Male | 3424 (2608,4435) | 18.12 (13.65,23.66) | 99926 (78568,122923) | 484.6 (373.92,624.54) |
|  | Both | 6399 (4794,8401) | 14.69 (10.96,19.31) | 140035 (117462,164539) | 380.26 (290.51,494.94) |
| Philippines | Female | 1425 (1151,1698) | 3.56 (2.87,4.22) | 13 (10,16) | 85.34 (69.02,102.49) |
|  | Male | 2525 (2004,3088) | 6.05 (4.85,7.3) | 32 (25,39) | 197.45 (156.48,242.31) |
|  | Both | 3950 (3370,4533) | 4.77 (4.06,5.49) | 45 (36,55) | 141.24 (119.31,163.95) |
| Seychelles | Female | 1 (1,1) | 0.91 (0.68,1.15) | 5520 (3769,7310) | 24.12 (19.03,29.66) |
|  | Male | 1 (1,1) | 1.70 (1.33,2.12) | 10894 (7569,14580) | 53.71 (42.15,65.63) |
|  | Both | 1 (1,2) | 1.32 (1.03,1.63) | 16414 (11503,21574) | 39.93 (31.45,48.44) |
| Sri Lanka | Female | 251 (172,330) | 1.81 (1.23,2.36) | 62191 (48334,77672) | 40.24 (27.79,53.01) |
|  | Male | 342 (233,458) | 3.24 (2.24,4.28) | 142897 (110311,180138) | 97.2 (68.53,128.87) |
|  | Both | 593 (409,775) | 2.46 (1.71,3.2) | 205088 (161546,254122) | 67.31 (47.63,87.82) |
| Thailand | Female | 2772 (2072,3519) | 4.94 (3.8,6.2) | 80 (52,110) | 133.04 (107.11,166.23) |
|  | Male | 4283 (3329,5356) | 10.38 (8.14,12.78) | 119 (82,156) | 392.61 (303.04,497.32) |
|  | Both | 7055 (5582,8818) | 7.55 (6,9.24) | 198 (136,270) | 259.2 (203.92,322.67) |
| Timor-Leste | Female | 2 (1,3) | 0.36 (0.17,0.55) | 73738 (43659,109692) | 11.98 (7.9,16.67) |
|  | Male | 2 (2,3) | 0.44 (0.25,0.68) | 140496 (85604,202812) | 16.96 (11.85,22.74) |
|  | Both | 4 (3,6) | 0.40 (0.21,0.62) | 214234 (132265,308740) | 14.48 (10.04,19.74) |
| Viet Nam | Female | 3428 (1794,5244) | 6.74 (3.52,10.26) | 2822063 (1970115,3763712) | 142.46 (85.89,210.52) |
|  | Male | 4635 (2574,7034) | 12.84 (6.84,19.46) | 3955221 (2708619,5148711) | 320.1 (196.17,465.01) |
|  | Both | 8062 (4398,12174) | 9.29 (4.99,14.02) | 214234 (132265,308740) | 224.38 (139.25,322.69) |

Abbreviations: DALYs, disability-adjusted life years

Table S2. The annual percent change (APC) and average annual percentage change (AAPC) of the age-standardized mortality rate (ASMR) attributed to high temperature in South Asia and Southeast Asia from 1990 to 2021 based on the joinpoint regression analysis model.

| **Location** | **Sex** | **Period** | **APC (95% CI)** | **AAPC (95% CI)** |
| --- | --- | --- | --- | --- |
| South Asia | Female | 1990-2019 | 0.34 (-0.29 to 8.39) | -0.67 (-1.53 to 0.72) |
|  |  | 2019-2021 | -14.21 (-25.32 to 0.14) |  |
|  | Male | 1990-2019 | 0.71* (0.17 to 8.13) | -0.27 (-1.14 to 1.05) |
|  |  | 2019-2021 | -13.52 (-24.47 to 0.45) |  |
| Bangladesh | Female | 1990-2021 | 0.45 (-0.42 to 1.34) | 0.45 (-0.42 to 1.34) |
|  | Male | 1990-2021 | 0.26 (-0.55 to 1.08) | 0.26 (-0.55 to 1.08) |
| Bhutan | Female | 1990-2021 | 0.36 (-0.32 to 1.06) | 0.36 (-0.32 to 1.06) |
|  | Male | 1990-2021 | 0.75***** (0.07 to 1.44) | 0.75***** (0.07 to 1.44) |
| India | Female | 1990-2021 | 0.15 (-0.48 to 0.8) | 0.15 (-0.48 to 0.8) |
|  | Male | 1990-2019 | 0.91***** (0.32 to 5.9) | -0.23 (-1.09 to 1.09) |
|  |  | 2019-2021 | -15.37 (-26.23 to 0.48) |  |
| Nepal | Female | 1990-2021 | -0.87 (-2 to 0.29) | -0.87 (-2 to 0.29) |
|  | Male | 1990-2021 | -0.10 (-1.22 to 1.03) | -0.10 (-1.22 to 1.03) |
| Pakistan | Female | 1990-2002 | 2.34***** (0.81 to 9.06) | 0.50 (-0.15 to 1.19) |
|  |  | 2002-2021 | -0.64 (-3.35 to 0.1) |  |
|  | Male | 1990-2002 | 2.76***** (1.21 to 7.13) | 0.68 ***** (0.07 to 1.3) |
|  |  | 2002-2021 | -0.61 (-2.49 to 0.14) |  |
| Southeast Asia | Female | 1990-2021 | 0.74 (-0.48 to 2) | 0.74 (-0.48 to 2) |
|  | Male | 1990-2021 | 0.60 (-0.34 to 1.57) | 0.60 (-0.34 to 1.57) |
| Cambodia | Female | 1990-2021 | 1.36 (-0.66 to 3.44) | 1.36 (-0.66 to 3.44) |
|  | Male | 1990-2021 | 1.59 (-0.29 to 3.5) | 1.59 (-0.29 to 3.5) |
| Indonesia | Female | 1990-2021 | 4.21***** (1.61 to 6.98) | 4.21***** (1.61 to 6.98) |
|  | Male | 1990-2021 | 3.69***** (1.6 to 5.88) | 3.69***** (1.6 to 5.88) |
| Lao People's Democratic Republic | Female | 1990-2021 | 0.71 (-0.99 to 2.46) | 0.71 (-0.99 to 2.46) |
|  | Male | 1990-2021 | 0.80 (-0.85 to 2.49) | 0.80 (-0.85 to 2.49) |
| Malaysia | Female | 1990-2021 | 4.02 (1.97 to 6.16) | 4.02* (1.97 to 6.16) |
|  | Male | 1990-2021 | 2.71 (1.12 to 4.33) | 2.71* (1.12 to 4.33) |
| Maldives | Female | 1990-2021 | -1.22 (-2.53 to 0.13) | -1.22 (-2.53 to 0.13) |
|  | Male | 1990-2021 | -0.58 (-1.67 to 0.53) | -0.58 (-1.67 to 0.53) |
| Mauritius | Female | 1990-2021 | 4.47***** (1.35 to 7.77) | 4.47* (1.35 to 7.77) |
|  | Male | 1990-2021 | 4.28***** (1 to 7.66) | 4.28* (1 to 7.66) |
| Myanmar | Female | 1990-2021 | -0.99 (-2.09 to 0.13) | -0.99 (-2.09 to 0.13) |
|  | Male | 1990-2021 | -0.30 (-1.27 to 0.71) | -0.30 (-1.27 to 0.71) |
| Philippines | Female | 1990-2021 | 3.03 (1.56 to 4.55) | 3.03* (1.56 to 4.55) |
|  | Male | 1990-2021 | 2.58 (1.37 to 3.82) | 2.58* (1.37 to 3.82) |
| Seychelles | Female | 1990-2021 | 2.53***** (0.82 to 4.23) | 2.53* (0.82 to 4.23) |
|  | Male | 1990-2021 | 0.45 (-0.89 to 1.82) | 0.45 (-0.89 to 1.82) |
| Sri Lanka | Female | 1990-2021 | 0.85 (-0.9 to 2.73) | 0.85 (-0.9 to 2.73) |
|  | Male | 1990-2021 | -0.09 (-1.21 to 1.08) | -0.09 (-1.21 to 1.08) |
| Thailand | Female | 1990-2021 | -0.46 (-1.84 to 0.9) | -0.46 (-1.84 to 0.9) |
|  | Male | 1990-2021 | -1.08***** (-2.11 to -0.05) | -1.08* (-2.11 to -0.05) |
| Timor-Leste | Female | 1990-2021 | 10.21***** (7.31 to 13.11) | 10.21* (7.31 to 13.11) |
|  | Male | 1990-2000 | 15.7* (-22.6 to -3) | 4.66* (2.09 to 8.68) |
|  |  | 2000-2003 | 81.94* (29 to 130.71) |  |
|  |  | 2003-2021 | 7.64* (2.67 to 10.65) |  |
| Viet Nam | Female | 1990-2021 | 1.38***** (0.45 to 2.35) | 1.38* (0.45 to 2.35) |
|  | Male | 1990-2021 | 1.61***** (0.73 to 2.54) | 1.61* (0.73 to 2.54) |

* Indicates *p*< 0.05. The equality of APC and AAPC values indicates the absence of trend inflection points (i.e., zero joinpoints) throughout the period. CI, confidence interval.

Table S3. The annual percent change (APC) and average annual percentage change (AAPC) of the age-standardized DALYs rate (ASDR) attributed to high temperature in South Asia and Southeast Asia from 1990 to 2021 based on the joinpoint regression analysis model.

| **Location** | **Sex** | **Period** | **APC (95% CI)** | **AAPC (95% CI)** |
| --- | --- | --- | --- | --- |
| South Asia | Female | 1990-2019 | -0.69 (-1.13 to 1.13) | -1.82*(-2.6 to -0.75) |
|  |  | 2019-2021 | -16.84(-26.55 to -1.44) |  |
|  | Male | 1990-2019 | -0.25(-0.65 to 2.03) | -1.23*(-1.92 to -0.26) |
|  |  | 2019-2021 | -14.36(-23.26 to -0.79) |  |
| Bangladesh | Female | 1990-2021 | -2.45*(-3.38 to -1.47) | -2.45*(-3.38 to -1.47) |
|  | Male | 1990-2021 | -2.37*(-3.07 to -1.65) | -2.37*(-3.07 to -1.65) |
| Bhutan | Female | 1990-2021 | -1.61*(-2.31 to -0.91) | -1.61*(-2.31 to -0.91) |
|  | Male | 1990-2021 | -1.08*(-1.72 to -0.41) | -1.08*(-1.72 to -0.41) |
| India | Female | 1990-2019 | -0.66(-1.11 to 0.07) | -2.01*(-2.8 to -1.19) |
|  |  | 2019-2021 | -19.71*(-29.53 to -3.04) |  |
|  | Male | 1990-2019 | -0.1(-0.56 to 2.67) | -1.25 *(-2.08 to -0.09) |
|  |  | 2019-2021 | -16.46*(-26.62 to -0.75) |  |
| Nepal | Female | 1990-2021 | -2.91*(-3.96 to -1.81) | -2.91*(-3.96 to -1.81) |
|  | Male | 1990-2021 | -2.13*(-3.14 to -1.1) | -2.13*(-3.14 to -1.1) |
| Pakistan | Female | 1990-2002 | 1.22(-0.16 to 11.46) | -0.17(-1.07 to 0.73) |
|  |  | 2002-2021 | -1.46*(-9.92 to -0.24) |  |
|  | Male | 1990-2002 | 1.57(0.33 to 6.97) | 0.05(-0.61 to 0.72) |
|  |  | 2002-2021 | -1.2*(-5.14 to -0.31) |  |
| Southeast Asia | Female | 1990-2021 | -0.63(-2.11 to 0.95) | -0.63(-2.11 to 0.95) |
|  | Male | 1990-2021 | -0.72(-1.66 to 0.26) | -0.72(-1.66 to 0.26) |
| Cambodia | Female | 1990-2021 | -0.51(-2.42 to 1.44) | -0.51(-2.42 to 1.44) |
|  | Male | 1990-2021 | -0.06(-1.67 to 1.6) | -0.06(-1.67 to 1.6) |
| Indonesia | Female | 1990-2021 | 2.1(-0.85 to 5.25) | 2.1(-0.85 to 5.25) |
|  | Male | 1990-2021 | 1.43(-0.71 to 3.68) | 1.43(-0.71 to 3.68) |
| Lao People's Democratic Republic | Female | 1990-2021 | -1.1(-2.65 to 0.5) | -1.1 (-2.65 to 0.5) |
|  | Male | 1990-2021 | -0.84(-2.28 to 0.62) | -0.84 (-2.28 to 0.62) |
| Malaysia | Female | 1990-2021 | 2.82*(1.09 to 4.59) | 2.82* (1.09 to 4.59) |
|  | Male | 1990-2021 | 1.88*(0.62 to 3.2) | 1.88* (0.62 to 3.2) |
| Maldives | Female | 1990-2021 | -2.5*(-3.69 to -1.24) | -2.5* (-3.69 to -1.24) |
|  | Male | 1990-2021 | -1.65*(-2.62 to -0.66) | -1.65* (-2.62 to -0.66) |
| Mauritius | Female | 1990-2021 | 4.46*(1.34 to 7.74) | 4.46* (1.34 to 7.74) |
|  | Male | 1990-2021 | 4.43*(1.15 to 7.82) | 4.43* (1.15 to 7.82) |
| Myanmar | Female | 1990-2021 | -2.11*(-3.57 to -0.59) | -2.11 *(-3.57 to -0.59) |
|  | Male | 1990-2021 | -1.34*(-2.4 to -0.24) | -1.34* (-2.4 to -0.24) |
| Philippines | Female | 1990-1996 | -9.61(-37.5 to 2.03) | 0.81 (-1.09 to 3.25) |
|  |  | 1996-2021 | 3.49*(1.45 to 14.74) |  |
|  | Male | 1990-1996 | -7.28(-30.33 to 1.06) | 0.42 (-1.1 to 2.18) |
|  |  | 1996-2021 | 2.36*(1.01 to 11.23) |  |
| Seychelles | Female | 1990-2021 | 1.67*(0.25 to 3.08) | 1.67*(0.25 to 3.08) |
|  | Male | 1990-2021 | 0.09(-1.17 to 1.38) | 0.09(-1.17 to 1.38) |
| Sri Lanka | Female | 1990-2021 | -0.6(-2.92 to 1.87) | -0.6(-2.92 to 1.87) |
|  | Male | 1990-2021 | -1.44*(-2.83 to 0) | -1.44*(-2.83 to 0) |
| Thailand | Female | 1990-2021 | -1.5*(-2.65 to -0.34) | -1.5*(-2.65 to -0.34) |
|  | Male | 1990-2005 | 0.63(-0.94 to 4.49) |  |
|  |  | 2005-2008 | -17.01*(-22.48 to -3.94) | -0.97*(-1.62 to -0.04) |
|  |  | 2008-2021 | 1.25(-0.78 to 6.44) |  |
| Timor-Leste | Female | 1990-1002 | -44.93(-60.99 to 2.71) | -1.36(-3.54 to 3.12) |
|  |  | 1992-2021 | 2.68(-17.45 to 16.86) |  |
|  | Male | 1990-2010 | -3.76*(-10.28 to -1.31) | 0.39(-1.67 to 2.27) |
|  |  | 2010-2021 | 8.41*(2.06 to 31.94) |  |
| Viet Nam | Female | 1990-2021 | 0.12(-0.76 to 1.04) | 0.12(-0.76 to 1.04) |
|  | Male | 1990-2021 | 0.32(-0.49 to 1.15) | 0.32(-0.49 to 1.15) |

* Indicates *p*< 0.05. The equality of APC and AAPC values indicates the absence of trend inflection points (i.e., zero joinpoints) throughout the period. CI, confidence interval.

- Appendix 3. Figure S1~S10

Fig S1. The rate of (A) death and (B) DALYs attributed to high temperature in different age groups and countries of South Asia and Southeast Asia in 2021.

Fig S2.Trends of the age-standardized mortality rate (ASMR) attributed to high temperature in different countries of South Asia and Southeast Asia from 1990 to 2021.

Fig S3.Trends of the age-standardized DALYs rate (ASDR) attributed to high temperature in different countries of South Asia and Southeast Asia from 1990 to 2021.

Fig S4. The different causes of mortality rate attributed to high temperature in children under 5 years old in South Asia and Southeast Asia in 2021.(A) Level 1 and (B) Level 2 causes.

Fig S5. The different causes of mortality rate attributed to high temperature in elderly over 55 years old in South Asia and Southeast Asia in 2021.(A) Level 1 and (B) Level 2 causes

Fig S6.The different level 2 causes of the age-standardized mortality rate (ASMR) attributed to high temperature in different countries of South Asia and Southeast Asia in 2021.

Fig S7.The different level 2 causes of the age-standardized DALYs rate (ASDR) attributed to high temperature in different countries of South Asia and Southeast Asia in 2021.

Fig S8. Age, period, and cohort effects on the DALYs attributed to high temperature in South Asia and Southeast Asia. (A-C) Age, period, and cohort effects, respectively.

Fig S9. Decomposition analysis of the age-standardized mortality rate (ASMR) and the age-standardized DALYs rate (ASDR) attributed to high temperature in different countries of (A) Southeast Asia and (B) South Asia.

Fig S10. Projects the age-standardized mortality rate (ASMR) attributed to high temperature in different countries of South Asia and Southeast Asia from 2025 to 2045.


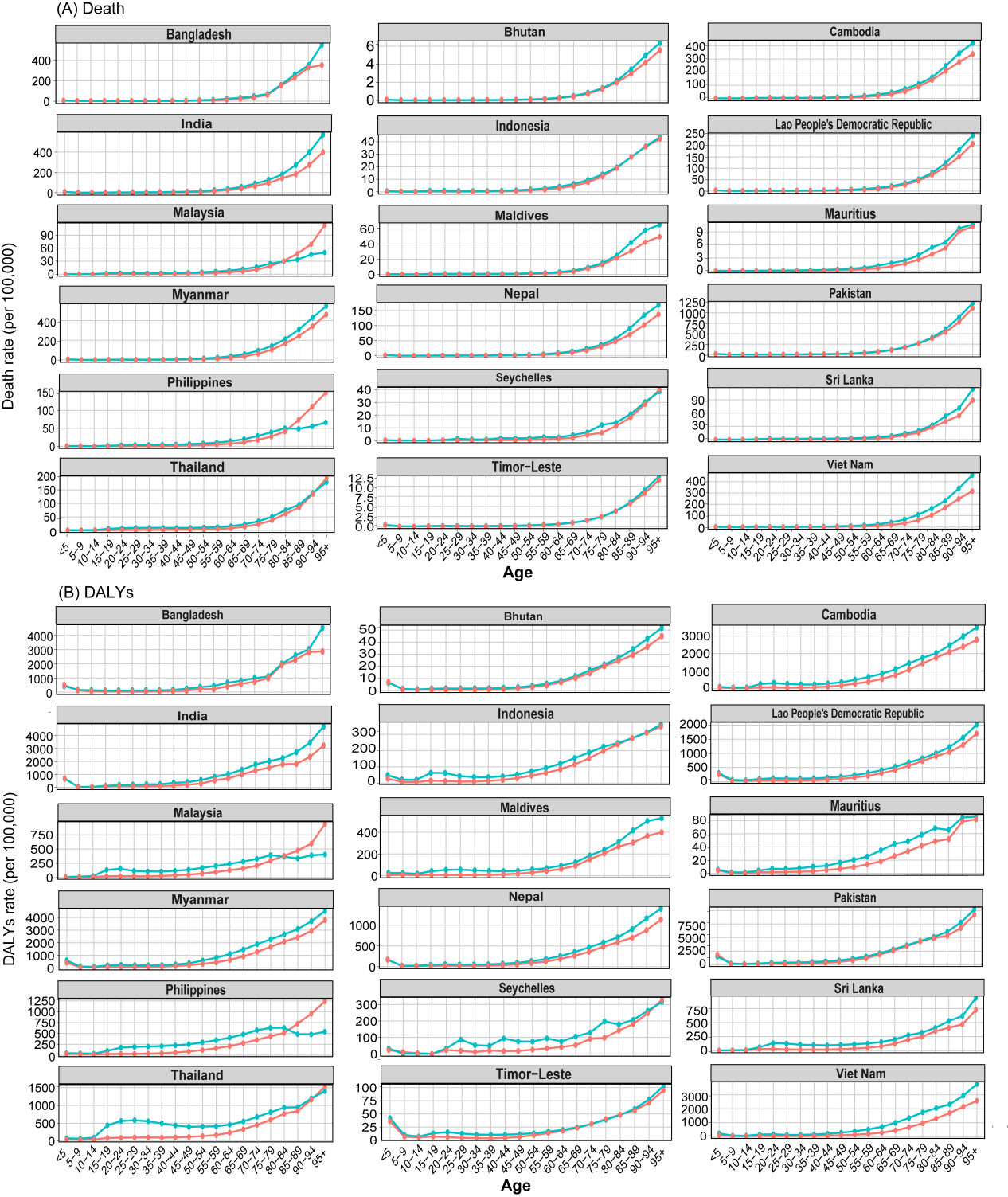


Fig S1. The rate of (A) death and (B) DALYs attributed to high temperature in different age groups and countries of South Asia and Southeast Asia in 2021.


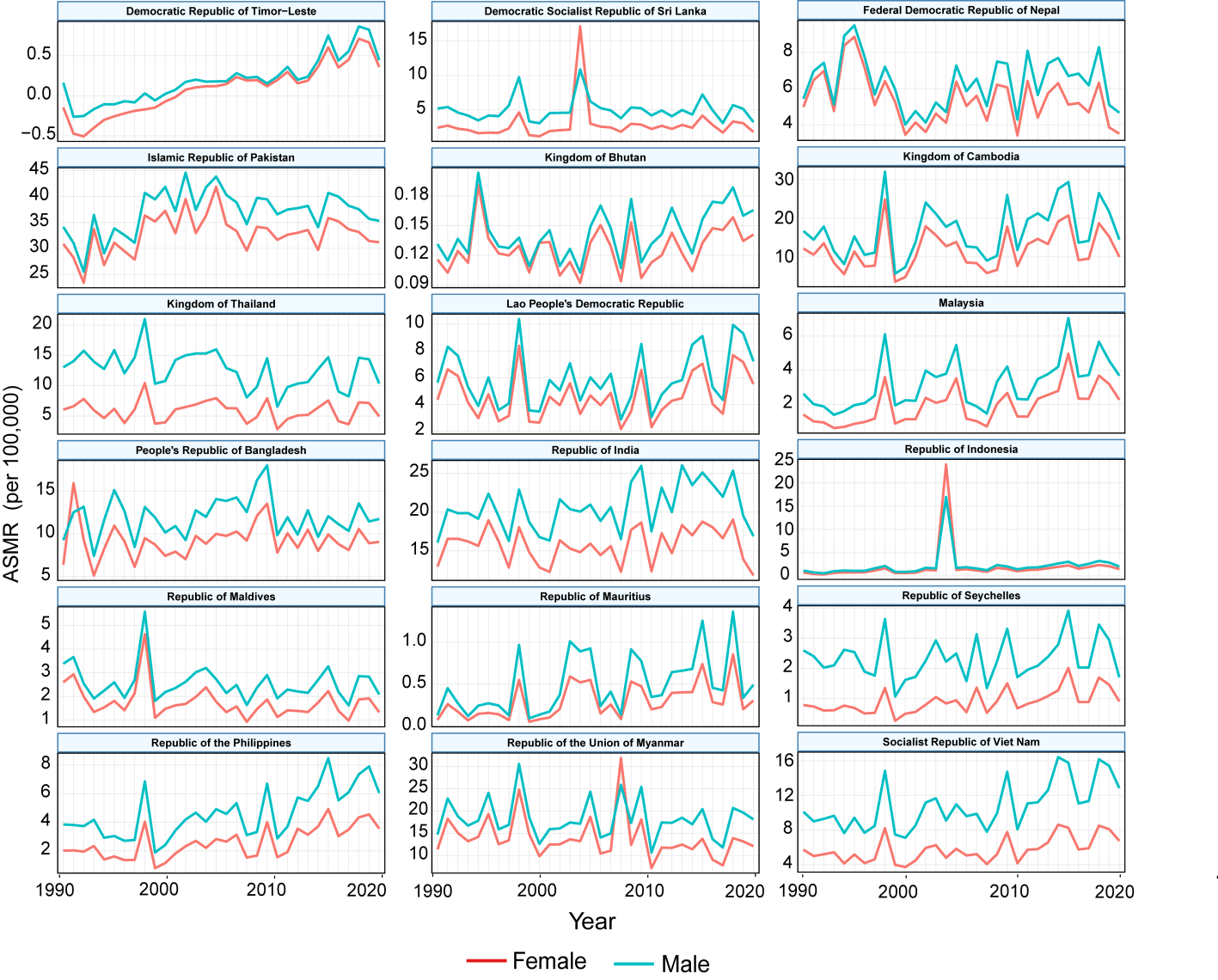


Fig S2.Trends of the age-standardized mortality rate (ASMR) attributed to high temperature in different countries of South Asia and Southeast Asia from 1990 to 2021.


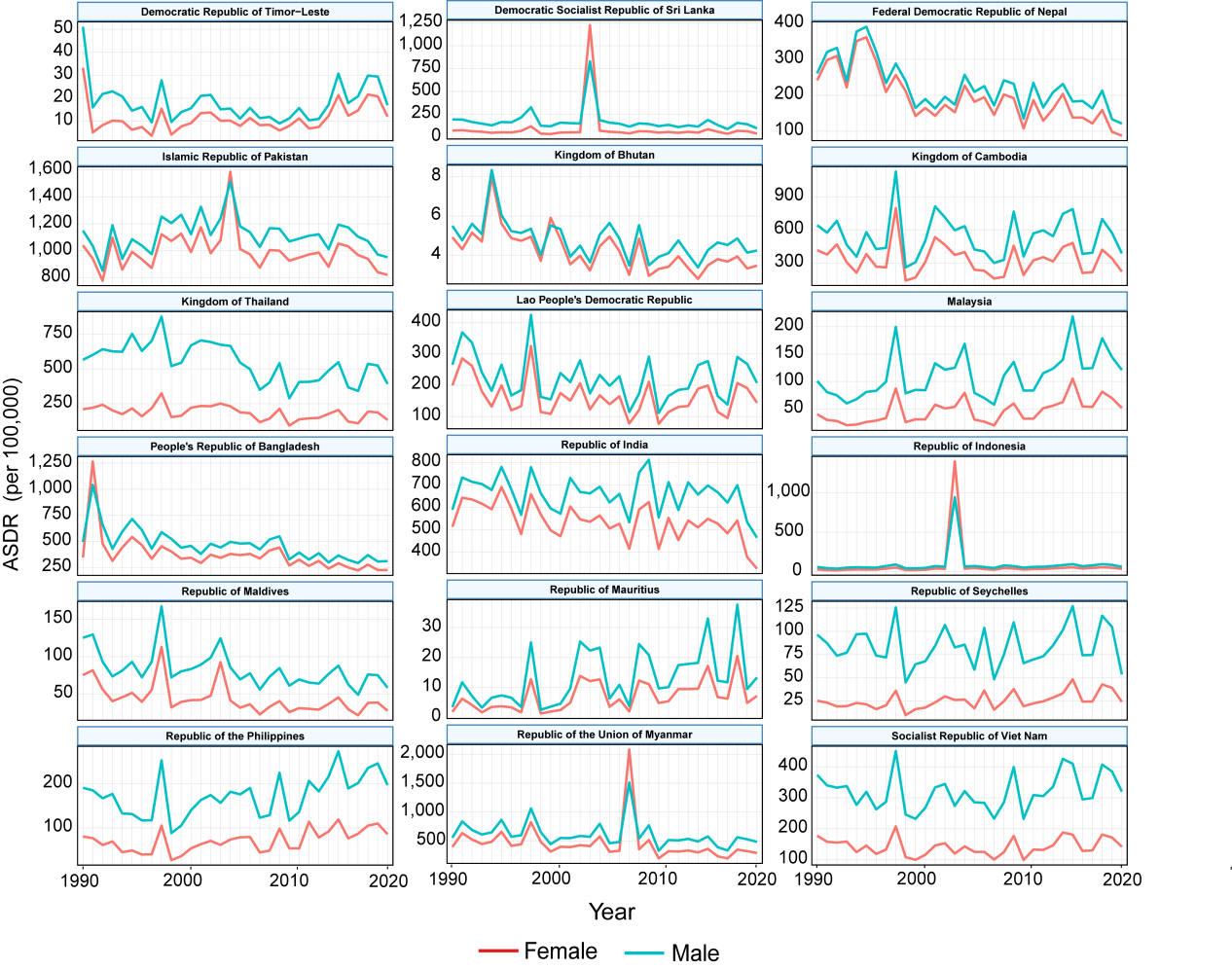


Fig S3.Trends of the age-standardized DALYs rate (ASDR) attributed to high temperature in different countries of South Asia and Southeast Asia from 1990 to 2021.


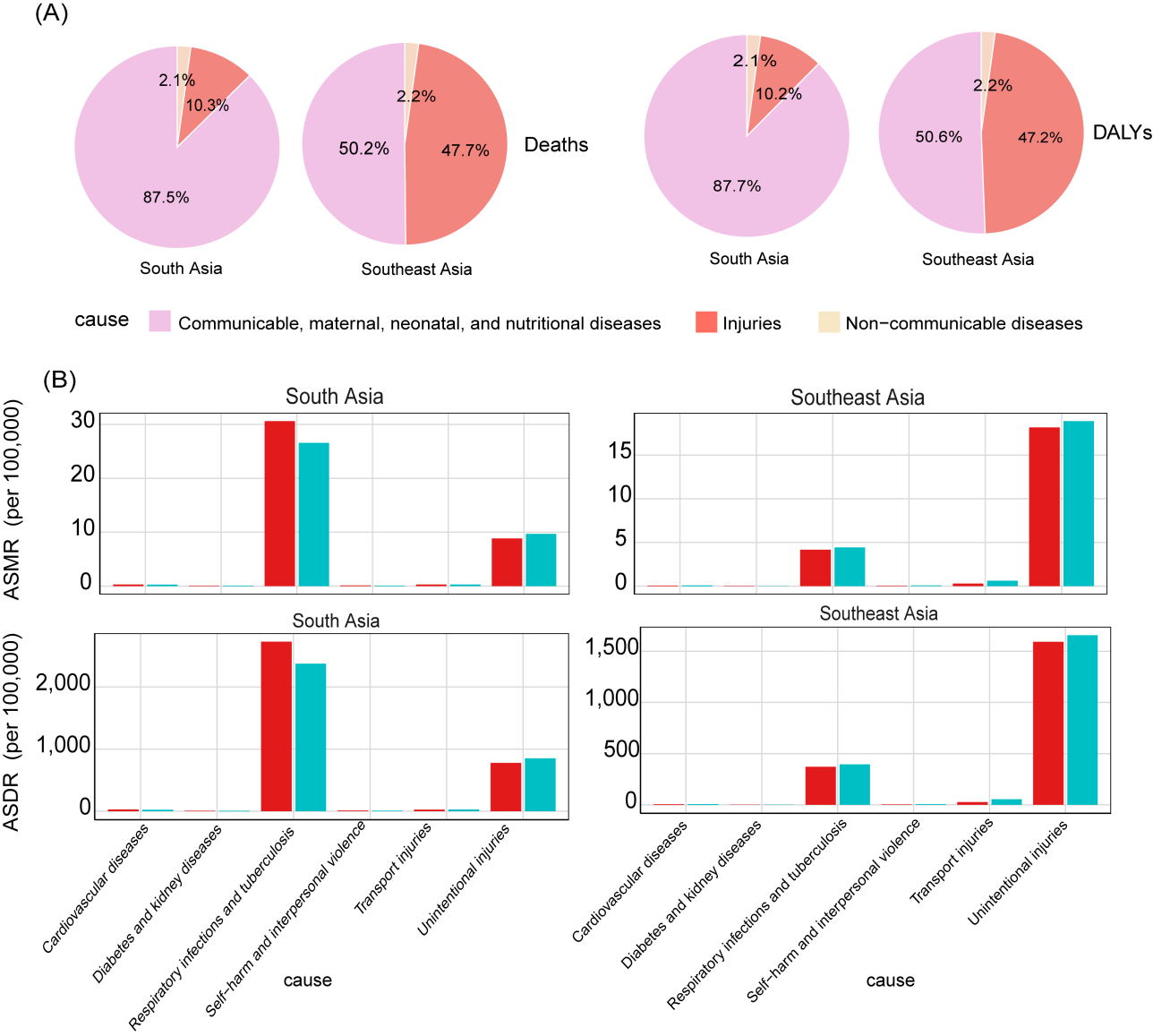


Fig S4. The different causes of mortality rate attributed to high temperature in children under 5 years old in South Asia and Southeast Asia in 2021.(A) Level 1 and (B) Level 2 causes.


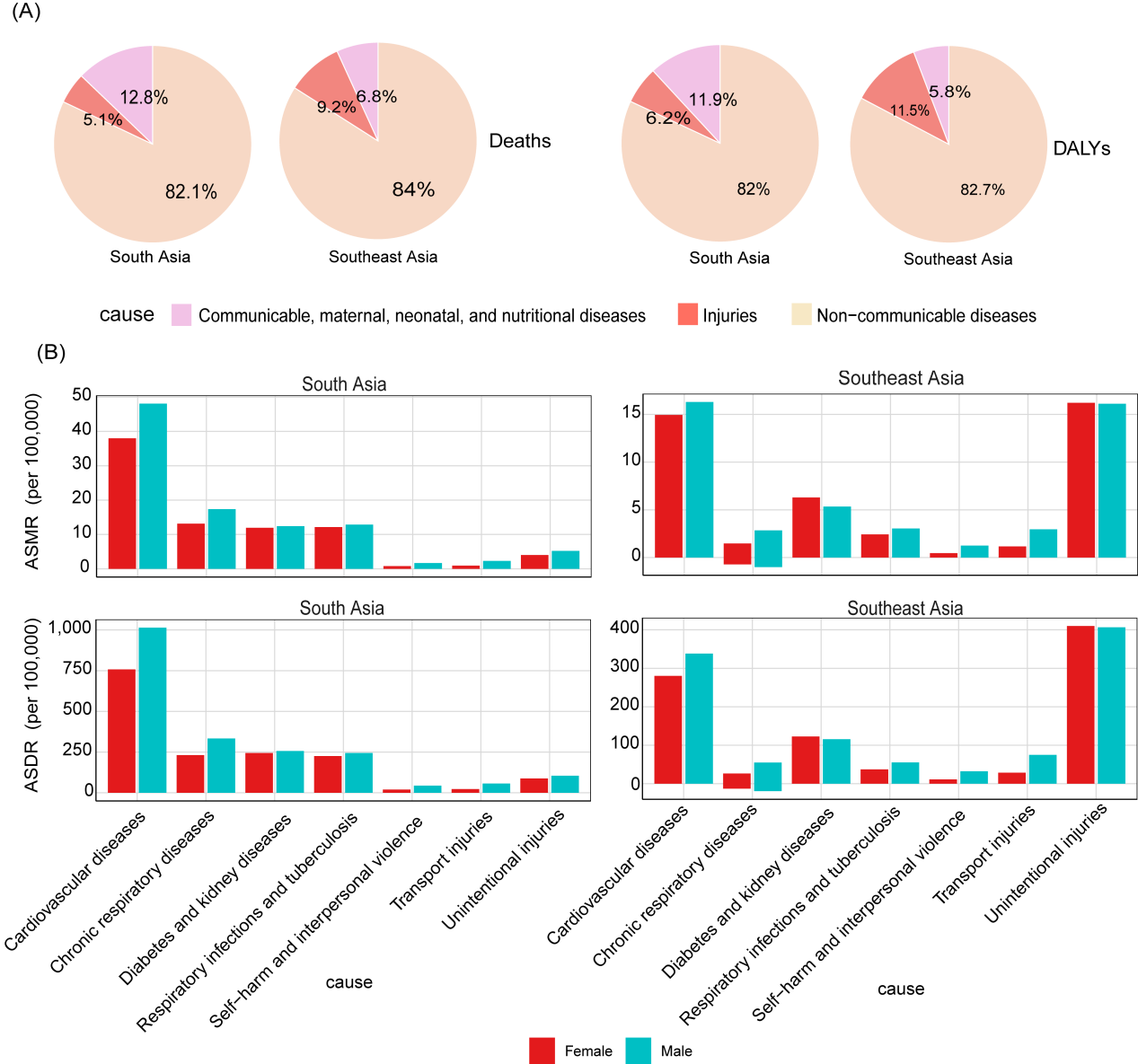


Fig S5. The different causes of mortality rate attributed to high temperature in elderly over 55 years old in South Asia and Southeast Asia in 2021.(A) Level 1 and (B) Level 2 causes.


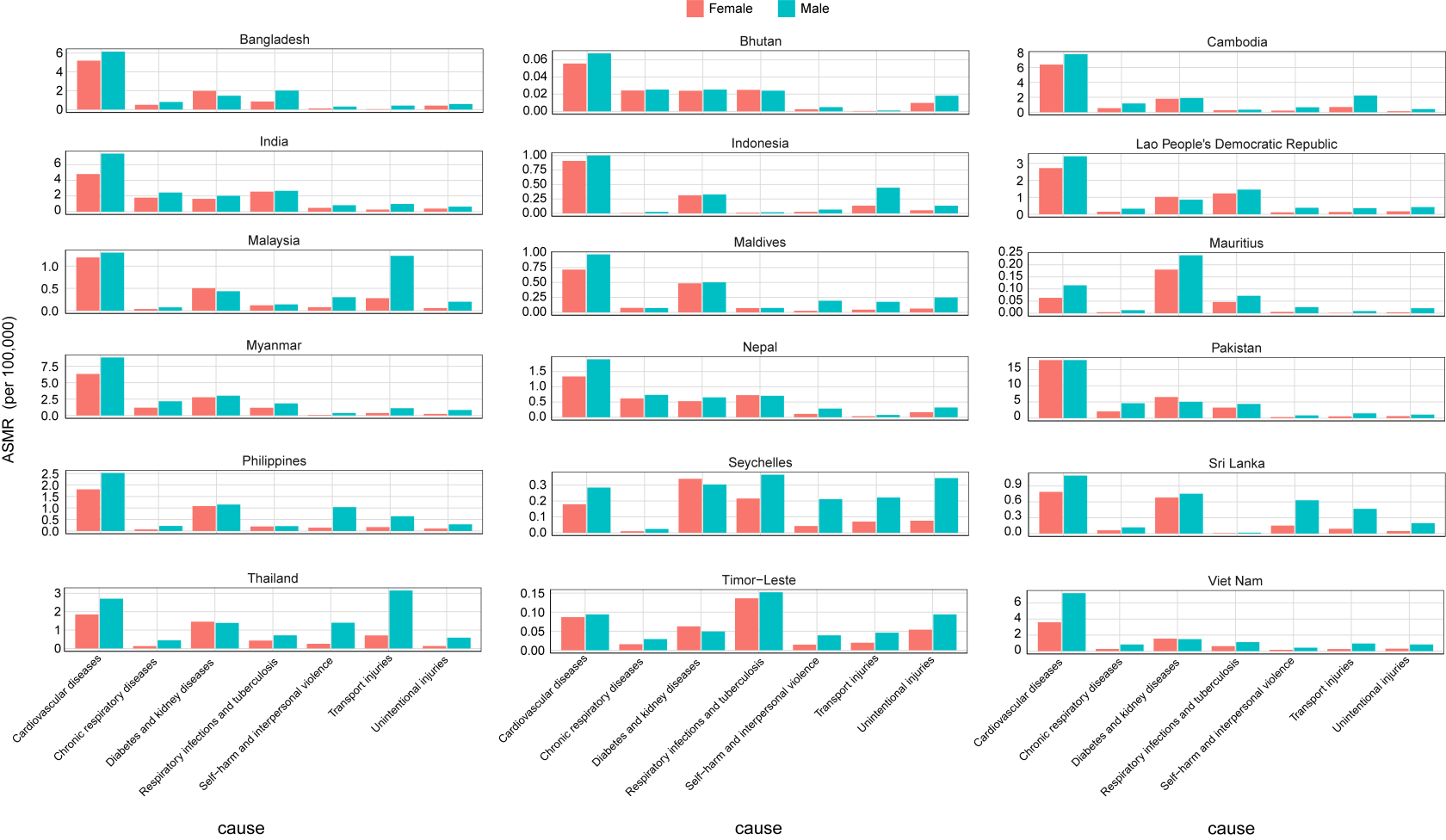


Fig S6.The different level 2 causes of the age-standardized mortality rate (ASMR) attributed to high temperature in different countries of South Asia and Southeast Asia in 2021.


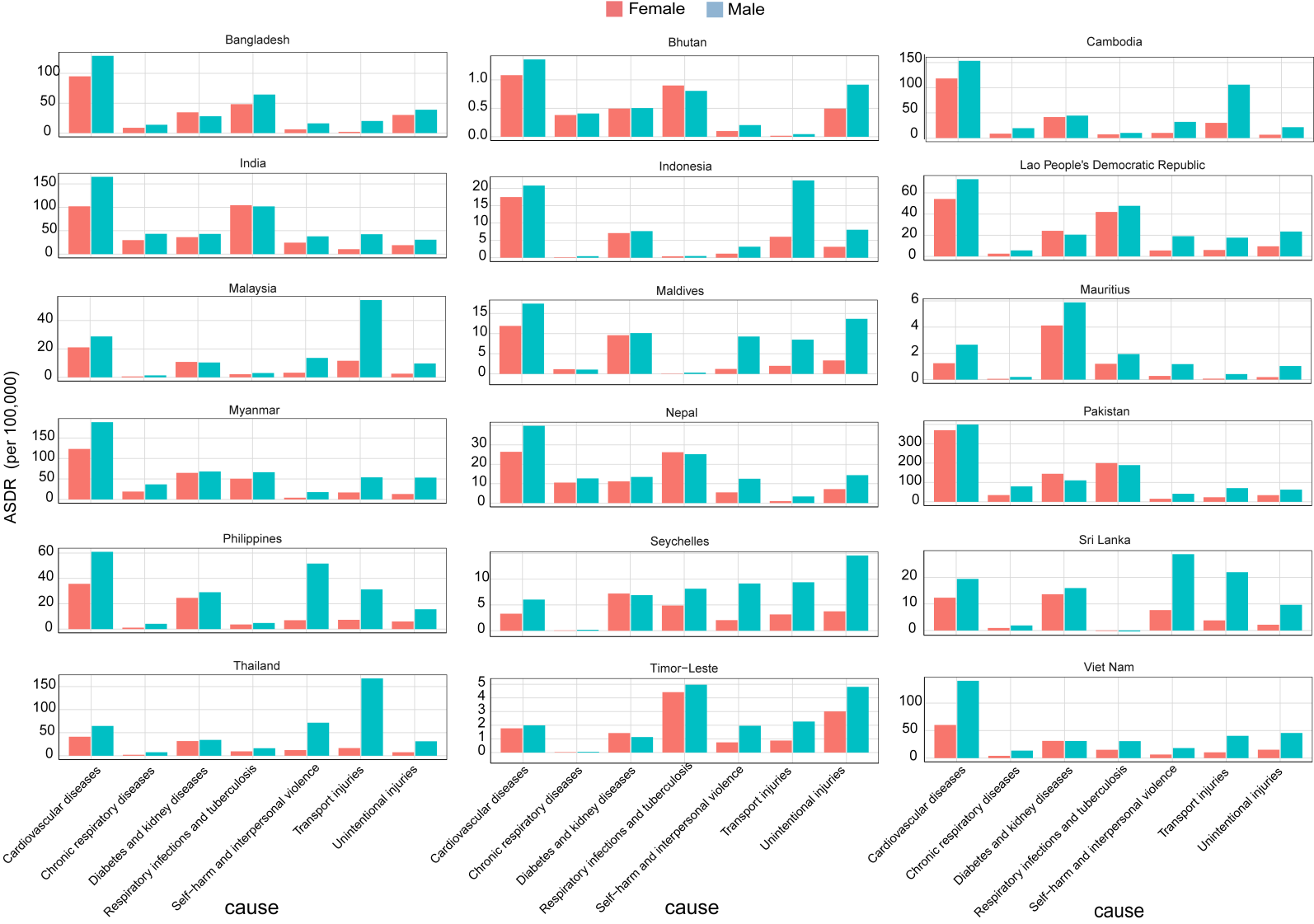


Fig S7.The different level 2 causes of the age-standardized DALYs rate (ASDR) attributed to high temperature in different countries of South Asia and Southeast Asia in 2021.


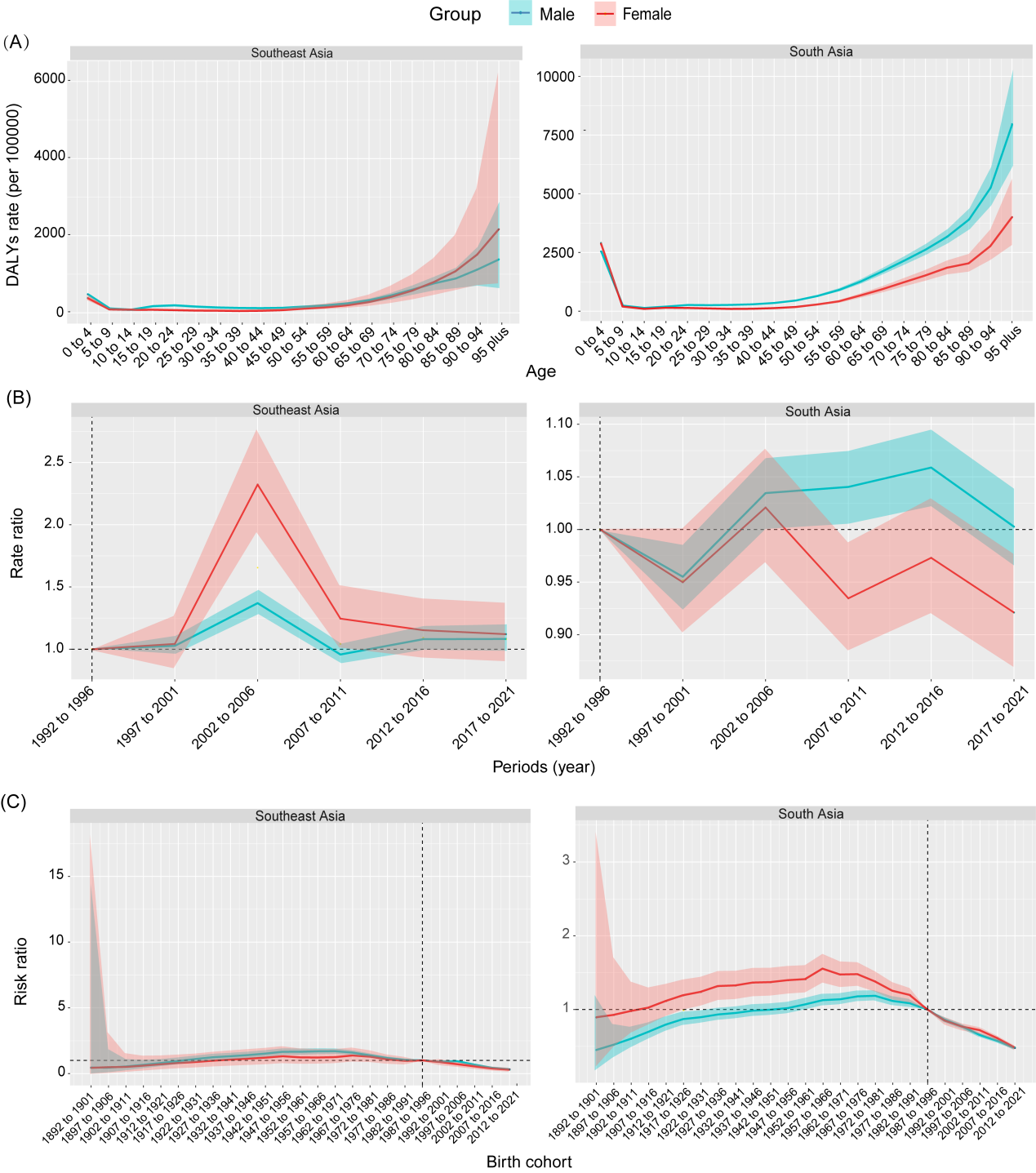


Fig S8. Age, period, and cohort effects on the DALYs attributed to high temperature in South Asia and Southeast Asia. (A-C) Age, period, and cohort effects, respectively.


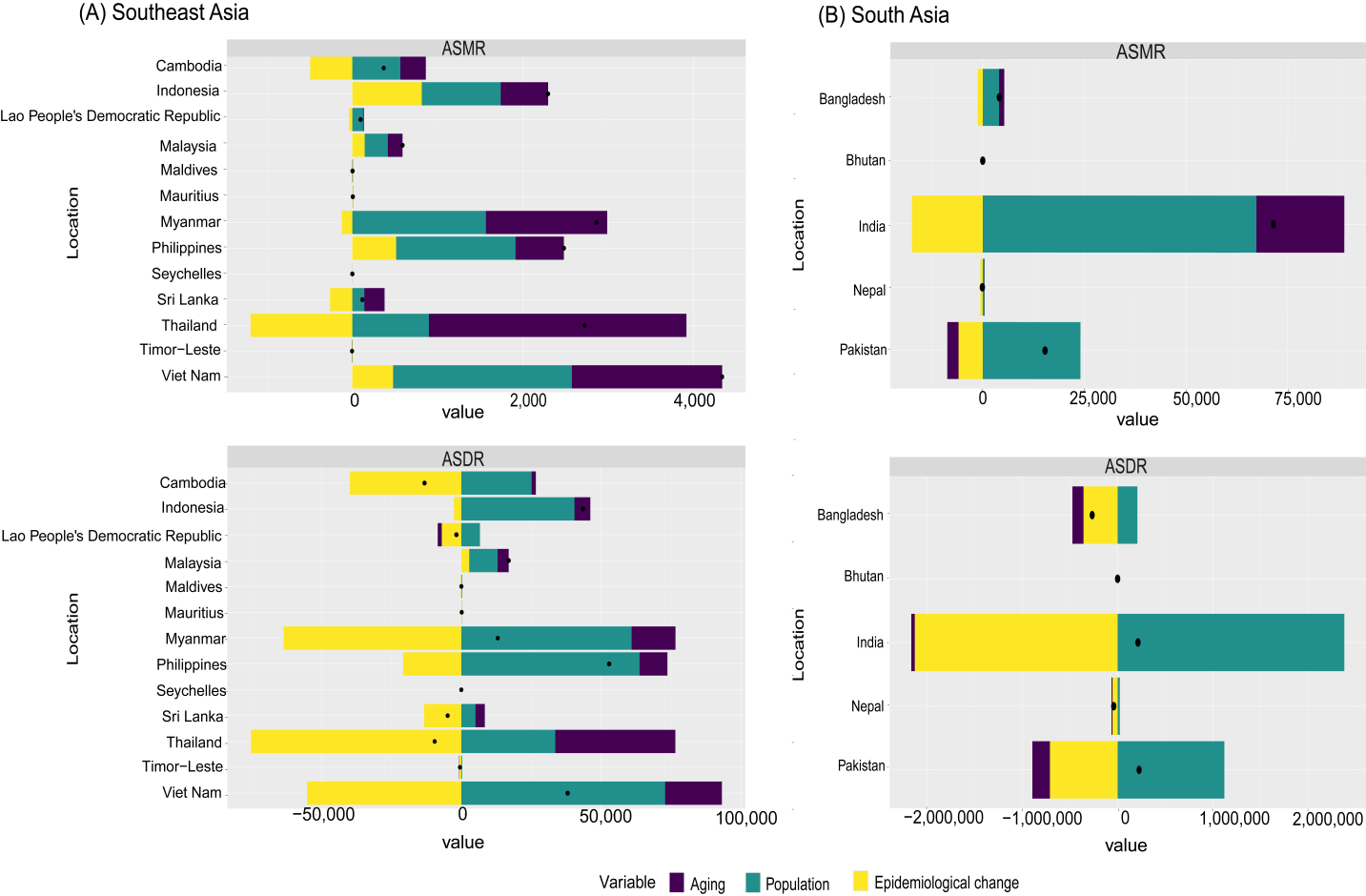


Fig S9. Decomposition analysis of the age-standardized mortality rate (ASMR) and the age-standardized DALYs rate (ASDR) attributed to high temperature in different countries of (A) Southeast Asia and (B) South Asia.


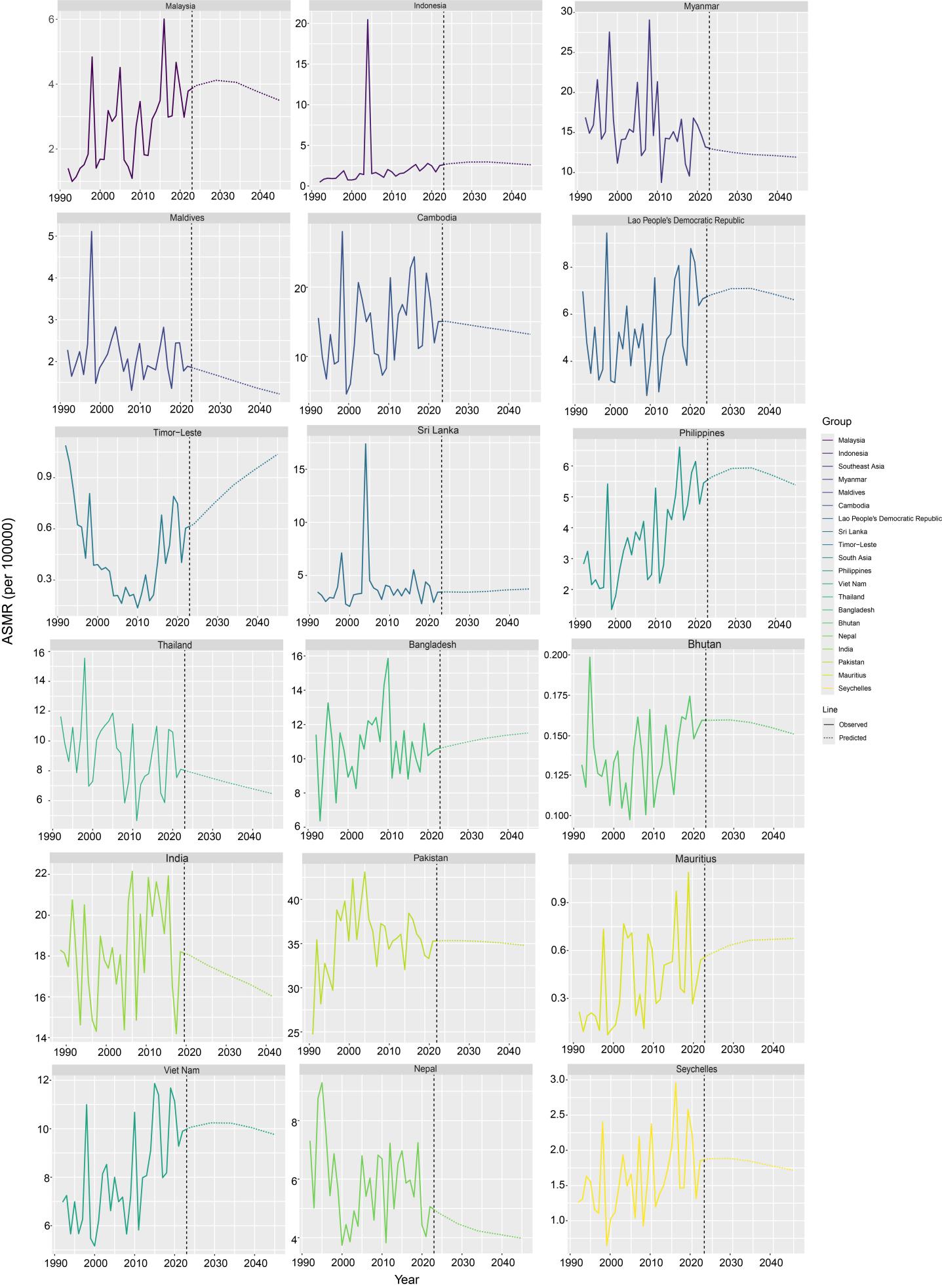


Fig S10. Projects the age-standardized mortality rate (ASMR) attributed to high temperature in different countries of South Asia and Southeast Asia from 2025 to 2045.
